# Synthesis and biophysical evaluation of carbosilane dendrimers as therapeutic siRNA carriers

**DOI:** 10.1038/s41598-024-51238-w

**Published:** 2024-01-18

**Authors:** Serafin Zawadzki, Ángela Martín-Serrano, Elżbieta Okła, Marta Kędzierska, Sandra Garcia-Gallego, Paula O. López, Francisco J. de la Mata, Sylwia Michlewska, Tomasz Makowski, Maksim Ionov, Elżbieta Pędziwiatr-Werbicka, Maria Bryszewska, Katarzyna Miłowska

**Affiliations:** 1 Department of General Biophysics, Faculty of Biology and Environmental Protection, University of Lodz, 141/143 Pomorska St., 90-236 Lodz, Poland; 2 BioMedChem Doctoral School of the University of Lodz and Lodz Institutes of the Polish Academy of Sciences, 21/23 Matejki St., 90-237 Lodz, Poland; 3 Department of Organic and Inorganic Chemistry, IQAR, University of Alcalá, 28805 Madrid, Spain; 4 Networking Research Center on Bioengineering, Biomaterials and Nanomedicine (CIBER-BBN), 28029 Madrid, Spain; 5 Ramón y Cajal Health Research Institute (IRYCIS), 28034 Madrid, Spain; 6 Laboratory of Microscopic Imaging and Specialized Biological Techniques, Faculty of Biology and Environmental Protection, University of Lodz, Banacha 12/16, 90-237 Lodz, Poland; 7 Centre of Molecular and Macromolecular Studies, Polish Academy of Sciences, Sienkiewicza 112, 90-363 Lodz, Poland; 8 Faculty of Medicine, Collegium Medicum, Mazovian Academy in Plock, 2 Dabrowskiego Sq, 09-402 Plock, Poland

**Keywords:** Nanoparticles, Dendrimers, Nanoscale biophysics

## Abstract

Gene therapy presents an innovative approach to the treatment of previously incurable diseases. The advancement of research in the field of nanotechnology has the potential to overcome the current limitations and challenges of conventional therapy methods, and therefore to unlocking the full potential of dendrimers for use in the gene therapy of neurodegenerative disorders. The blood–brain barrier (BBB) poses a significant challenge when delivering therapeutic agents to the central nervous system. In this study, we investigated the biophysical properties of dendrimers and their complexes with siRNA directed against the apolipoprotein E (APOE) gene to identify an appropriate nanocarrier capable of safely delivering the cargo across the BBB. Our study yielded valuable insights into the complexation process, stability over time, the mechanisms of interaction, the influence of dendrimers on the oligonucleotide's spatial structure, and the potential cytotoxic effects on human cerebral microvascular endothelium cells. Based on our findings, we identified that the dendrimer G3Si PEG6000 was an optimal candidate for further research, potentially serving as a nanocarrier capable of safely delivering therapeutic agents across the BBB for the treatment of neurodegenerative disorders.

## Introduction

It was nearly a century ago when the British bacteriologist Griffith described the process of bacterial transformation and almost 5 decades later, Friedmann and Roblin- the precursors of modern genomics technology, hypothesized the idea of delivering an exogenous gene copy for the treatment of genetically inherited diseases^1,2^. Today, gene therapy provides curative alternatives for disorders that were previously untreatable. Most modern, conventional therapies target proteins with the aim of symptomatic relief, but do not address underlying pathologies. Employing nucleic acids may modify cell gene expression obtaining therapeutic effects for the treatment of many diseases such as cancers, cardiovascular disorders, infectious diseases, ocular, inflammatory diseases and neurological pathologies^3^.

Gene therapy was originally defined to be the use of DNA in the treatment of various illnesses^4^. As science and technology progresses, more strategies have emerged which now allow cDNA, rDNA, mRNA, ASO, miRNA, siRNA and restriction enzymes to be used as potential medicinal agents^5^. The strategies utilizing these listed medical agents may be divided generally into: gene correction (replacement or repair of a malfunctioning gene), augmentation (introduction of a functional version of a mutant), inactivation (silencing the expression of an abnormal gene) and suicide gene therapy (implementation of a gene to a cancer cell that expresses an enzyme that can convert pro-drugs into cytotoxic agents)^6^.

To achieve the desired therapeutic result an extraordinary engineering challenge must be solved. Since naked nucleic acid implementations are largely inefficient for their limited resilience to enzymatic activity, intrinsic stability, and inability to penetrate lipid bilayers, it is necessary to design an effective delivery vector^7^. Attaining successful gene functionality requires the vector to transport and unload the delivered therapeutics into target cells efficiently while not causing side effects. It must prevent cargo degradation and clearance, evade immune detection and prevent nonspecific interactions. Moreover, the vectors must have large-scale production and commercialization properties. The intended effect can be achieved by both viral vectors and non-viral vectors. Selecting the appropriate vector depends on the duration of expression of the transported material, its size, as well as the intended destination^8^. Both viral and non-viral vectors have been used in clinical trials and have unique benefits. Although viruses have evolved to cross multiple anatomical barriers and efficiently transfer their genomes to mammalian cells, their utilization in gene therapy is also associated with several drawbacks. Viral vectors exhibit intrinsic disadvantages, such as high immunogenicity, that led to the inflammatory reactions that caused the first gene therapy-related fatality due to treatment with an adenovirus vector^9^. Another significant detrimental effect concerning the usage of viral vectors is their potential carcinogenetic effect, low specificity, low packaging capacity and high production cost^8,10^.

These limitations have prompted researchers to look for new strategies for effective and safe transgene carriers. There is little to no doubt that non-viral vectors offer a significantly safer approach, including the decreased ability to undergo insertional mutagenesis, a lower pathogenicity and immunogenicity^11,12^. Additionally, they provide a variety of possible benefits, such as the ease of production, lower cost, higher stability and fewer restrictions on vector size^12^. However, initially, non-viral nanocarriers appeared to be less effective than viral approaches, but with subsequent developments they have increased their gene delivery efficiency.

Possible gene nanocarriers include many types of nanoparticles such as inorganic or metal nanoparticles, quantum dots, liposomes, micelles and dendrimers. Among these nanosystems, dendrimers have emerged as promising synthetic vectors since their first application as transfection agents^13^. Carbosilane dendrimers achieve good gene transfection efficiency in vitro, reaching values as high as the lentiviruses^14^. Several factors set dendrimers apart from other polymers, dendrimers being radially symmetrical nanoparticles with well-defined, homogeneous and monodisperse structures. Unlike stochastic polymers, dendrimers are produced methodically step-by-step. Their synthesis process controls their architecture and therefore their properties, such as shape, size, charge and solubility. Dendrimers consist of a symmetrical core from which radiating monomer units grow, forming branches known as “dendrons”. The repeated branching cycle is called a generation. Dendrimers are composed of inner and outer shells with many functional groups responsible for their high solubility and reactivity. This design by encapsulation, complexation or conjugation, allows dendrimers to carry different agents such as drug molecules, genetic material, targeting agents and dyes. Combining dendrimers with drugs or nucleic acids can improve treatment outcomes by increasing the solubility of the therapeutics, modifying pharmacokinetics and improving bioavailability. These features permit the delivery and application of medical agents that otherwise would not have a chance to be utilized^15,16^.

The dendrimer–nucleic acid complexes are called dendriplexes and are formed by electrostatic interactions. The interaction highly depends on the protonation of the dendrimer cationic groups. The strong interaction between the highly charged groups of the dendrimer and the gene causes wrapping of the DNA around the dendrimer, resulting in nucleosome-like structures^17^.

Positively charged dendriplexes increase enzymatic resistance and enhance cellular uptake, thereby increasing their transport into cells. Formation of colloidally stable, positively charged dendriplexes occurs when an excess of dendrimer exists in its non-complexed form. The ratio of nucleic acid to the nanoparticle is a crucial parameter in the formation of the complex. The free dendrimers also play a role in the internalization of the dendriplexes. Free dendrimers can significantly affect transfection efficiency and toxicity as the free polymer causes transient pore formation in the cell membrane, increasing permeability but also leading to cytotoxic side effects by disrupting the cellular membrane via nanohole formation, membrane thinning and erosion^18–20^.

Given their adjustable characteristics impacting pharmacokinetics, bioavailability, immunogenicity, toxicity, and transfection efficiency, dendrimers emerge as promising agents for gene delivery. Despite these promising features, a significant knowledge gap exists regarding the impact of dendrimer exposure in delivering biologically active compounds to the brain. In light of this, it becomes imperative to systematically design and investigate dendrimer nanocarriers delivering therapeutics across the most formidable barrier in the human body—the blood–brain barrier (BBB). Our study's objective is to evaluate the biophysical properties of dendrimers and their complexes with siRNA for the selection of a plausible candidate for the siRNA nanocarrier through the model of the blood–brain barrier. For this purpose, we assessed whether 4 carbosilane dendrimers could form complexes with siRNA directed against the apolipoprotein E (APOE) gene. The results of our investigation provided valuable insights into the complexation process, stability, the mechanisms of interaction, the influence of tested dendrimers on the oligonucleotide's spatial structure and possible cytotoxic effects.

siRNA directed against the APOE4 gene was selected for this study as the literature data indicates APOE4 as a factor in many disorders observed in Alzheimer’s disease, including disturbance of insulin signalling pathways in the brain, abnormal distribution of cholesterol and fatty acids, reduction of the integrity of the BBB and a decrease in glucose uptake by the brain^21^.

## Material and methods

### Dendrimers

The 2 types carbosilane dendrimers were synthesized in the Department of Organic and Inorganic Chemistry, University of Alcalá, Madrid, Spain:

1. Dendrimer with a silicon atom core, surface tertiary ammonium groups and polyethylene glycol (PEG) (one; 6000) -G3Si
2. Dendrimers with phloroglucinol core, surface tertiary ammonium groups (third or fourth generation) and PEG (one; 2000 or 6000)—G3O3 and G4O3.

**Figure 1:**
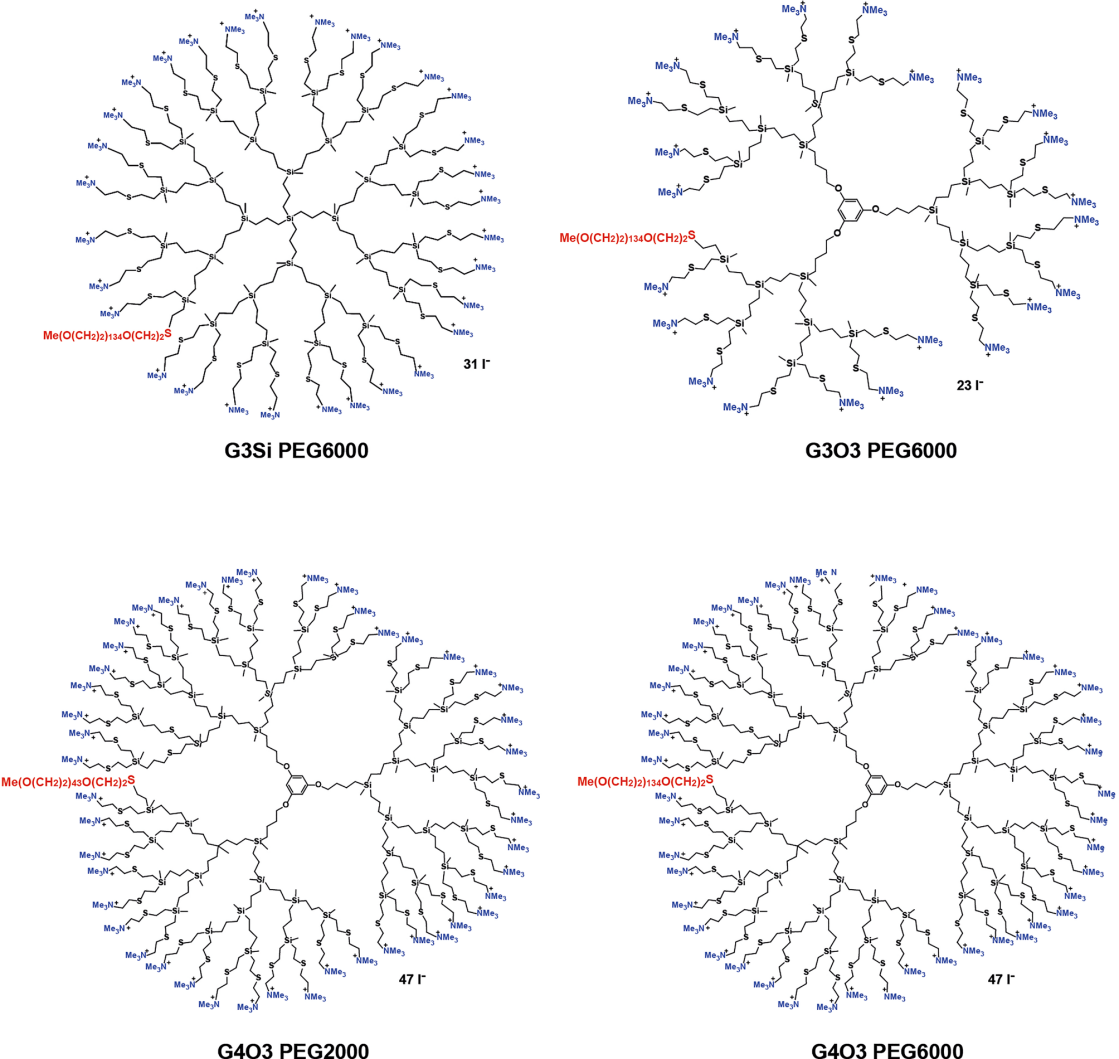
Structures of studied dendrimers.

Dendrimers are presented in Fig. 1 and Table 1.

**Table 1:** Chemical formula and molecular mass of the dendrimers.

Dendrimer	Abbreviation	Chemical formula	MW (g/mol)
G3Si(PEG6000)(NMe_3_^+^)_31_	G3Si PEG6000	C_601_H_1324_I_30_N_31_O_135_S_32_Si_29_	16,794.81
G3O3(PEG6000)(NMe_3_^+^)_23_	G3O3 PEG6000	C_527_H_1136_I_23_N_23_O_138_S_24_Si_21_	14,285.12
G4O3(PEG2000)(NMe_3_^+^)_47_	G4O3 PEG2000	C_609_H_1393_I_47_N_47_O_47_S_50_Si_44_	18,932.37
G4O3(PEG6000)(NMe_3_^+^)_47_	G4O3 PEG6000	C_790_H_1755_I_47_N_47_O_138_S_50_Si_44_	22,927.17

The choice of carbosilane as the dendrimer skeleton for our investigation stems from its distinctive attributes, characterized by low polarity and the high energy inherent in the C–Si bond. This structural framework confers heightened hydrophobicity relative to alternative dendrimer systems. The functionalization process applied to these dendrimers is executed in a statistically controlled manner, strategically introducing a singular polyethylene glycol (PEG) chain per dendritic molecule. This deliberate modification serves a dual purpose in our design strategy. Firstly, the inclusion of PEG chains is intended to mitigate potential toxicity associated with cationic charge derived from quaternary amines functioning as exterior terminal groups, enhancing the overall biocompatibility of the delivery system. Secondly, the modulation of the dendrimer's surface with PEG chains is anticipated to exert a nuanced influence on the strength of its electrostatic interactions with biomolecules such as serum proteins and prolong retention time in the circulatory system.

#### G_n_YV_m_

Precursor dendrimers with vinyl groups at the periphery and with a silicon (G_n_SiV_m_) or phloroglucinol (G_n_O_3_V_m_) core were synthesized as previously described in the literature^22,23^.

#### G_n_Y(SPEG)(SNMe_2_HCl)_m−1_

Corresponding precursor dendrimer G_n_YV_m_ (0.01 mmol), corresponding thiolated PEG (0.01 mmol) and 2,2-dimethoxy-2-phenylacetophenone (DMPA) (0.001 mmol) were dissolved in a 1:2 THF–methanol as solvent. After purging with argon for deoxygenation, the reaction mixture was irradiated for 1 h. After PEG incorporation, the rest of vinyl groups on the precursor dendrimers were functionalised as follows. 2-(dimethylamino)ethanethiol hydrochloride (0.00525·m-1 mmol) and DMPA (0.001 mmol) were added, the solution was purged with argon and irradiated for 1 h. This step was performed twice. The absence of vinyl groups after hydrothiolation was confirmed by ^1^H-NMR.

#### G_n_Y(SPEG)(SNMe_2_)_m−1_

Na_2_CO_3_ (0.01·m^−1^ mmol) was added to the reaction mixture containing corresponding G_n_Y(SPEG)(SNMe_2_HCl)_m−1_ dendrimer .The mixture was left to react overnight at room temperature. After complete neutralization was confirmed by ^1^H-NMR, the solvents were eliminated by rotaevaporation. Then THF was added and the solution was filtered to remove formed salts and DMPA.

#### G_n_Y(SPEG)(SNMe_3_I)_m−1_

Quaternization was performed by adding MeI (0.012·m mmol) to G_n_Y(SPEG)(SNMe_2_)_m-1_ solution in THF and left to react overnight at room temperature. Solvent and the excess of MeI were evaporated under reduced pressure. The final quaternized compounds were dissolved in water and purified by dialysis to remove excess of free thiol.

### siRNA

The following non-fluorescent or FITC-labelled siRNA was used in the present study (Dharmacon Inc, Lafayette, CO, USA):

Sense: 5′-GAUUACCUGCGCUGGGUGCUU.

Antisense: 5′-P GCACCCAGCGCAGGUAAUCUU.

### Other reagents

The HBEC-5i cell line derived from human cerebral microvascular endothelium (ATCC^®^ CRL3245™) was purchased from American Type Culture Collection ATCC^®^ (Manassas, VA, USA). DMEM-F12 (Dulbecco’s Modified Eagle Medium-F12) medium supplemented with stable Glutamine and 15 mM Hepes, along with Fetal bovine serum (FBS), were obtained from Biowest, Nuaillé, France. Sigma-Aldrich (Saint Louis, MO, USA) supplied Phosphate buffered saline (PBS) tablets, 1% Penicillin–Streptomycin, Endothelial cell growth supplement (ECGS) sourced from bovine neural tissue, and trypsin. Avantor (Radnor, PA, USA) supplied Dimethyl sulfoxide (DMSO) and 3-(4,5-2-yl)-2-5-diphenyl tetrazolium bromide (MTT). Maximus, (Łódź, Poland) provided Agarose, while Biotium, Inc. (Fremont, CA, USA) supplied GelRed stain. Other basic chemical reagents (sodium dihydrogen phosphate, disodium hydrogen phosphate) were obtained from the local supplier, Chempur (Piekary Śląskie, Poland). These chemicals were of analytical grade, and solutions were prepared using water purified using the Mili-Q system.

### Hydrodynamic diameter and zeta potential

The hydrodynamic diameter and zeta potential of the dendrimers and their complexes with siRNA were measured using Zetasizer Nano ZS, Malvern Instruments (UK). The dendrimers and dendriplexes were prepared in distilled water at room temperature. Increasing concentrations of the dendrimer in the range of 0.5–20 µM were added to siRNA at 0.5 µM and the zeta potential was measured. Each sample was measured 5 times in 7 runs. Zeta potential was calculated using the Helmholtz-Smoluchowski equation in Malvern software. The hydrodynamic diameter of the particles and their complexes were measured using dynamic light scattering (DLS), and the same concentrations of the siRNA and dendrimers as in the zeta potential measurement. Single samples were measured 5 times in 3 runs. Values with PDI > 0.5 were dismissed.

### Transmission electron microscopy (TEM)

The morphological structure, shape and size of the G3Si PEG6000 dendrimer was evaluated using a JEOL-1010 (Japan) transmission electron microscope at 100 000 × magnification. The dendriplex was created by combining siRNA with the dendrimer in PBS at room temperature. The dendriplex was prepared in a molar ratio of 2.5:1 (the optimal dendrimer/siRNA ratio), with a concentration of siRNA of 25 µM. After 20 min. incubation, 15 µl of the dendriplex or non-complexed dendrimer was placed for 10 min. on a 200-mesh copper grid with a carbon-coated surface. A saturated solution of uranyl acetate 2% (m/v) was used to stain both samples for 20 min. To enhance legibility, images have been sharpened.

### Atomic force microscopy (AFM)

The morphology studies of siRNA and dendrimers were conducted on droplets deposited on mica (Grade V-1 Muscovite) by atomic force microscopy (AFM). Measurements were carried out using the Flex Axiom Nanosurf apparatus with a C3000 controller (NanosurfAG, Liestal, Switzerland). The experiments were performed in the dynamic force mode (taping mode) and phase imaging mode. The analysis was performed using probes (HI’RES‐C14/CR‐AU μmasch) with a typical curve radius < 1 nm, a spring constant of 5 N/m and a resonance frequency of 160 kHz. The images were recorded with a resolution of 512 × 512 data points. Image analysis was carried out using Scanning Probe Image Processor Software (SPIP) by Image Metrology, Hørsholm, Denmark.

### Fluorescence polarization and time stability

This technique is based on the changes of fluorescence polarization of fluorescein labeled siRNA, after adding a dendrimer. The tested dendrimers were subsequently titrated to the siRNA-FITC solutions (1 μM, in 10 mM phosphate buffer) in order to achieve dendrimer/siRNA molar ratios ranging from 1:1 to 20:1. The dendrimer/siRNA mixtures were incubated at room temperature for 20 min. to ensure the formation of complexes, and then fluorescence polarization was measured on a Perkin-Elmer LS-55 spectrofluorometer in 1-cm path length quartz cuvettes. The excitation and emission wavelengths were 485 and 520 nm, with the excitation and emission slit widths being set to 7 and 5 nm, respectively. The cuvette holder was temperature controlled (25 °C).

Fluorescence polarization technique was also used to analyze the stability of the dendriplexes (dendrimer/siRNA in optimal saturation) in time. Measurements were taken immediately following a 20-min. pre-incubation period, as well as at 1, 5, 15 and 30-min. and subsequently at 1, 1.5, 2, 3, and 4-h later.

### Circular dichroism (CD)

Circular dichroism (CD) of siRNA (1 µM) titrated with increasing concentrations (0.5–10 µM) of dendrimers was measured with a Jasco J-815 CD spectrometer (Jasco International Co., Ltd., Tokyo, Japan) in 5-mm path length quartz cuvettes at 25 °C, with a wavelength step of 1 nm, a response time of 4 s and a scan rate of 50 nm/min. CD spectra for siRNA alone and in presence of dendrimers was measured between 200 and 320 nm. All samples were prepared in 10 mM phosphate buffer. Pure phosphate buffer was used also as a baseline.

### Gel electrophoresis

Electrophoresis in agarose gel was performed for the dendrimer/siRNA complexes. Dendrimers (0.5–10 µM) were mixed with siRNA (1 µM) in 10 mM Na-phosphate buffer, pH 7.4. and incubated at room temperature for 20 min. In the negative control, the non-complexed dendrimer was used at the maximum concentration tested. Meanwhile, in the positive control the siRNA was used without any dendrimer. Electrophoresis was run in a 3% agarose gel with GelRed stain (0.05 µg/ml) at 35 mA, 90 V for 45 min. The obtained electropherograms were analyzed using a ChemiDoc-It^2^ Imager (UVP, Cambridge, UK).

### Cell line

In vitro experiments were conducted with a HBEC-5i cell line (Human Brain Endothelial Cells). The cells were cultured on flasks covered with 1% gelatin in DMEM-F12 medium enriched with 1% Penicillin–Streptomycin, 10% FBS and 40 µg/ml ECGS under standard conditions (37 °C, 5% CO2).

### Cytotoxicity of dendrimers

HBEC-5i cells were seeded into 96-well plates at 1 × 10^4^ cells/well. Dendrimers were added to cells in increasing concentrations between 0.1 and 50 µM and incubated for 24 h (37 °C, 5% CO_2_). After incubation, cells were washed in PBS and incubated with 0.5 mg/mL of MTT at 37 °C for 3 h. Then the MTT was discarded carefully, and DMSO was added to solubilize the formazan crystals^24^. Finally, the absorbance was measured for each well at a wavelength of 570 nm using a Bio-Tek Synergy HT Microplate Reader (Bio-Tek Instruments, Winooski, VT, USA). All experiments were performed in 6 repetitions. Cell viability was calculated as the percent ratio of absorbance of the samples to the reference control, according to the formula:

$$\% \;{\text{viability}} = \left( {{\text{As}}/{\text{Ac}}} \right) \times 100\%$$

where As—absorbance of cells treated with dendrimer, Ac—absorbance of control cells (untreated).

### Statistical analysis

The results are presented as mean values ± standard deviation (SD). Segmental regression analysis was used to determine the ratio of the tested dendrimers to siRNA at which optimal saturation of the complex occurs.

## Results

### Dendrimer synthesis

**Figure 2:**
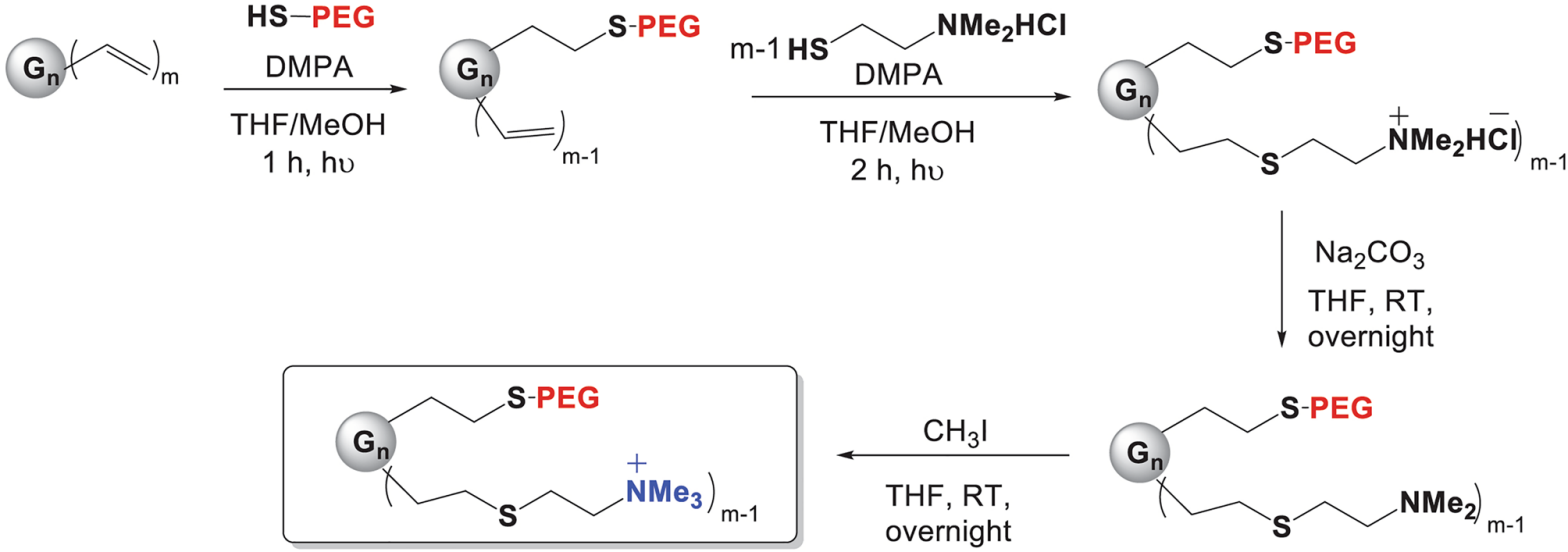
Synthesis of cationic pegylated dendrimers. “n” refers to dendrimer generation, “m” refers to number of peripheral functions. The thiolene addition reaction was employed to functionalize vinyl-terminated carbosilane dendrimers with alkene groups at the periphery. Commercial SH-PEG_x_ (x = 2000 or 6000) was used to decorate one branch, while 2-(Dimethylamino)ethanethiol hydrochloride was added to the remaining branches in the presence of DMPA as the photoinitiator. Neutralization with Na_2_CO_3_ and subsequent quaternization with MeI resulted in the synthesis of hetero-functionalized cationic pegylated carbosilane dendrimers with 23, 31, or 47 positive charges.

To carry out this work, we prepared 4 carbosilane pegylated cationic dendrimers to study the influence of dendritic core size as well as a number of cationic groups and pegylation, for biomedical purposes (Fig. 2). Heterofunctionalization with 2 types of commercial thiols, followed by neutralization and quaternization created G_n_Y(SPEG)(SNMe_3_I)_m−1_ dendrimers bearing PEG2000 or PEG6000 and 23 (G3O3), 31 (G3Si) or 47 (G4O3) positive charges (Fig. 6A). These compounds were perfectly soluble in water.

**Figure 3:**
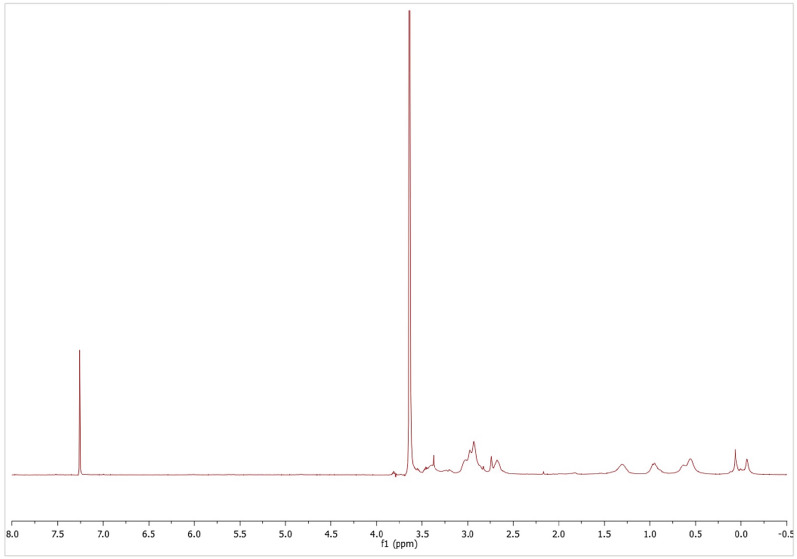
^1^H-NMR (CDCl_3_) of intermediate compound G_3_Si(SPEG6000)(SNMe_2_HCl)_31_. The signal at 3.60 ppm corresponds to PEG and NMe_2·_HCl, which are overlapped.

**Figure 4:**
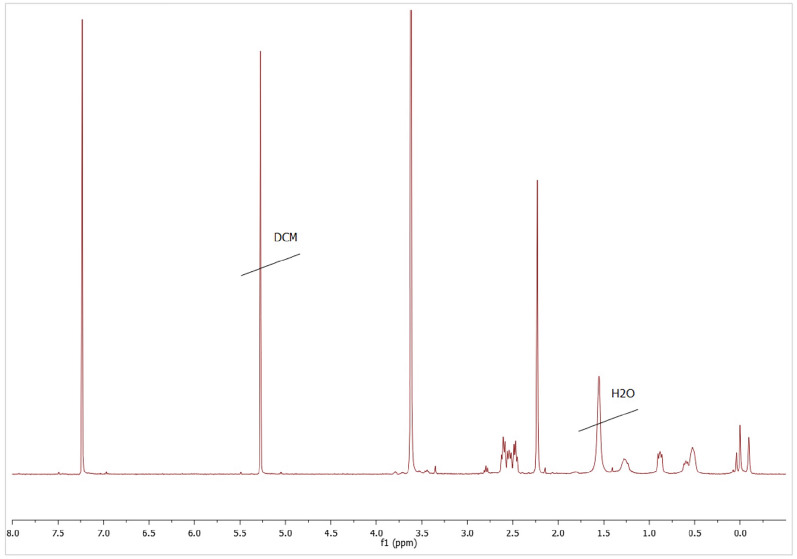
^1^H-NMR (CDCl_3_) of intermediate compound G_3_Si(SPEG6000)(SNMe_2_)_31_. The signal at 3.60 ppm corresponds to PEG and the signal at 2.25 ppm corresponds to NMe_2_.

**Figure 5:**
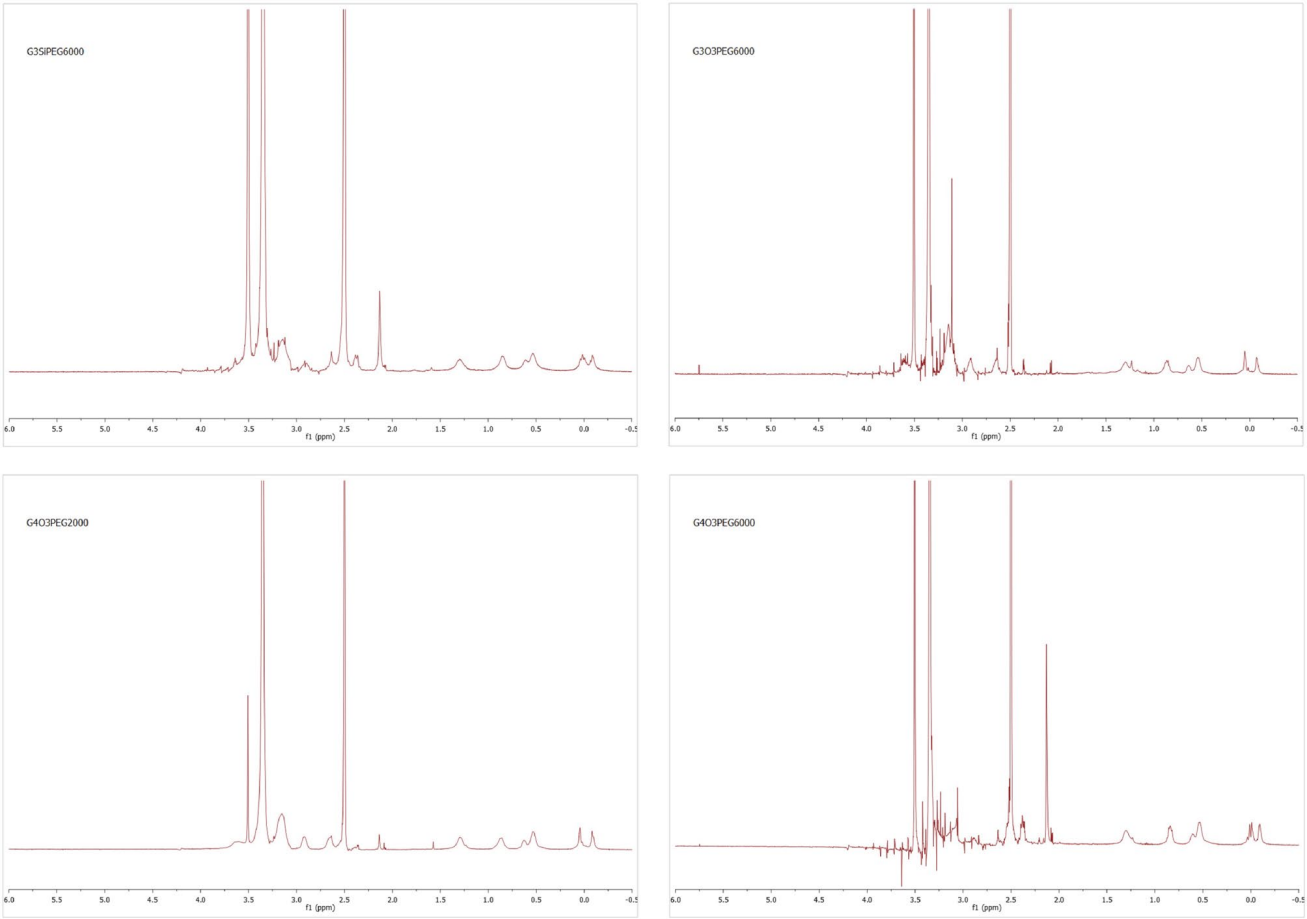
^1^H-NMR (DMSO) of the final compounds. The signal at 3.40 ppm corresponds to PEG and NMe_3_, which are overlapped.

Transformation of the vinyl precursor dendrimers into pegylated cationic analogues was followed by ^1^H-NMR. After PEG incorporation, a singlet appeared around 3.50 ppm, and vinyl representative signals at 6.15 ppm, 6.00 ppm and 5.71 ppm disappeared after the thiolene reaction with 2-(dimethylamino)ethanethiol hydrochloride was completed (Fig. 3). A representative singlet signal in G_n_Y(SPEG)(SNMe_2_)_m−1_ compounds at 2.25 ppm, which corresponded to neutralized peripheral dimethylamino groups disappearing by the time the reaction was completed (Fig. 4). Furthermore, methyl groups bound to the quaternary nitrogen atom appeared as a singlet around 3.40 ppm in G_n_Y(SPEG)(SNMe_3_I)_m−1_ compounds, overlapping with the PEG signal (Fig. 5). Detailed NMR spectra peak characterization can be found in supplementary Information.

### Zeta potential, size and morphological structure

The zeta potential and hydrodynamic diameter data for the tested dendrimers are presented in Table 2. The results indicate that all the dendrimers possess a positively charged surface in line with their chemical structures. Dendrimers G3O3 PEG6000 and G4O3 PEG6000 displayed the highest zeta potential, while the lowest values were observed for G3Si PEG6000 and G4O3 PEG2000.

**Table 2:** Zeta potential and hydrodynamic diameter of uncomplexed dendrimers (without siRNA).

Dendrimer	Z-potential (mV)	Z-size (nm)
G3Si PEG6000	20.23 ± 3.00	115.68 ± 57.10
G3O3 PEG6000	35.75 ± 2.60	261.26 ± 114.64
G4O3 PEG2000	25.83 ± 3.84	319.83 ± 108.23
G4O3 PEG6000	34.50 ± 2.86	248.22 ± 92.98

The data given from 3 repetitions as mean and standard deviation.

The hydrodynamic diameter, which incorporated both the core size of the nanoparticle and the surface molecules or polymers on the dendrimers, was determined via DLS particle size measurements. Based on these measurements, the dendrimer G4O3 PEG2000 displayed the largest hydrodynamic diameter, while G3Si PEG6000 exhibited the smallest.

**Figure 6:**
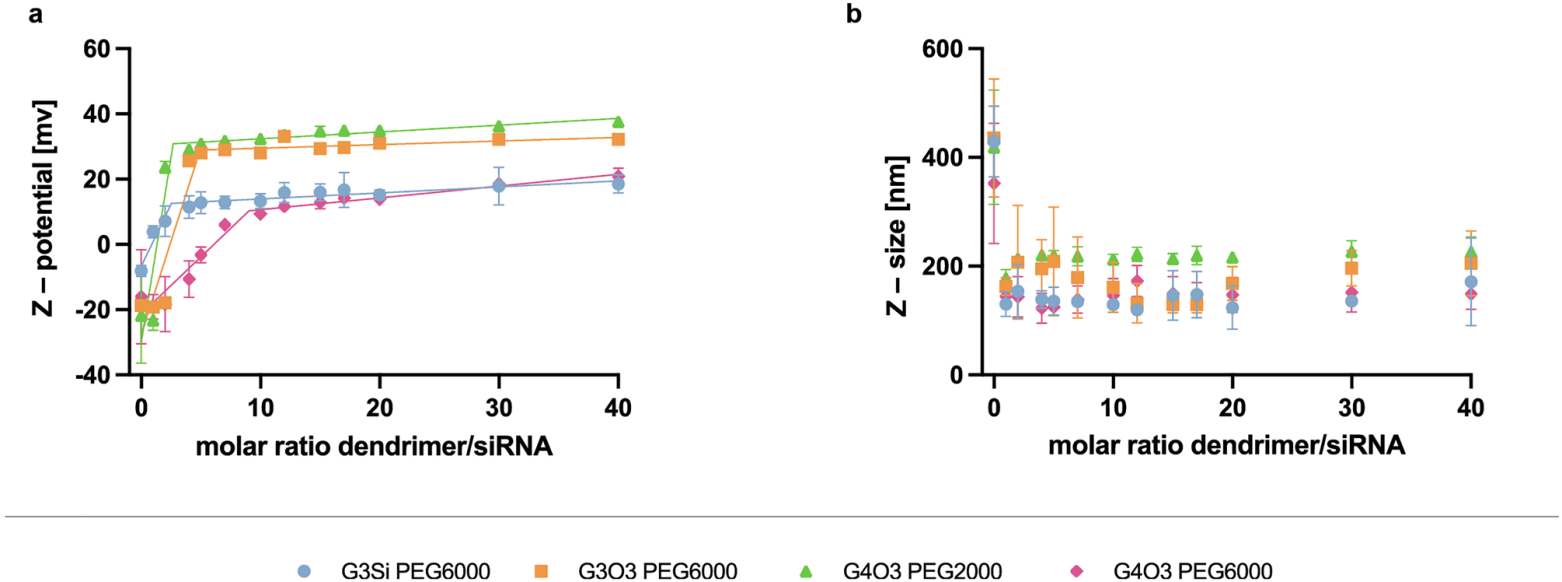
Zeta potential (**a**) and hydrodynamic diameter (**b**) of the dendrimer/siRNA complexes. The concentration of siRNA = 0.5 µM. The results are shown for 5 repetitions as mean and standard deviation.

Introducing the tested dendrimers to the siRNA led to a considerable increase in the zeta potential of siRNA (Fig. 6A). The G4O3 PEG2000/siRNA complex showed the highest zeta potential among all the studied dendriplexes. Notably, the continuous addition of dendrimer molecules to the siRNA complex reached a plateau, indicating a saturation point and suggesting that this is the optimal quantity of dendrimer molecules bound to the siRNA molecule.

The hydrodynamic size of the dendrimer/siRNA complexes, as determined by DLS, remained consistent regardless of dendrimer generation or concentration (Fig. 6B). The observed size range of the complexes was 100–330 nm. Increasing the dendrimer concentration has a negligible effect on the size of the complexes.

**Figure 7:**
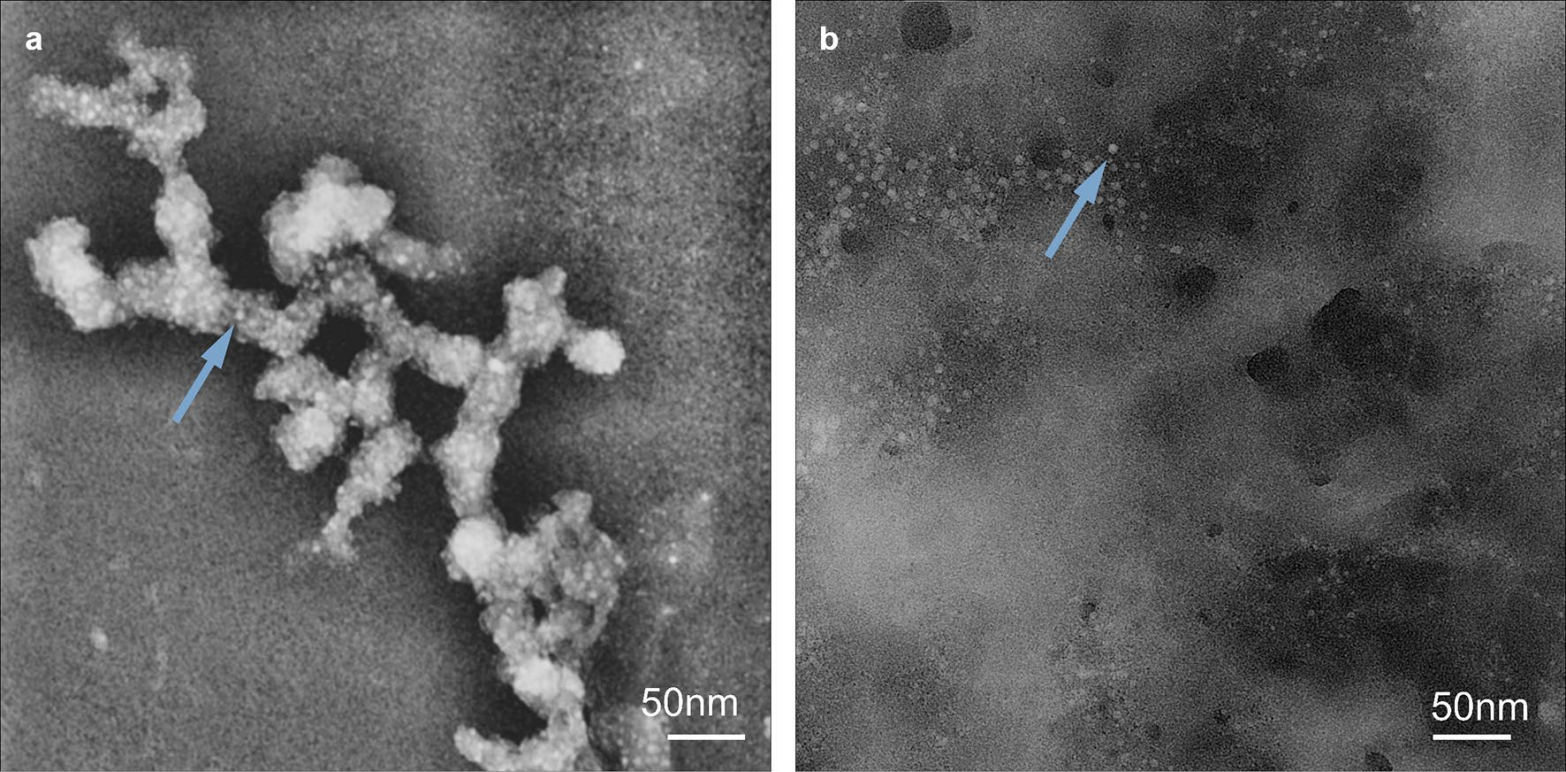
Transmission electron microscope images of the G3Si PEG6000 dendrimer (**a**) and G3Si PEG6000/siRNA dendriplex in a molar ratio of 2.5:1 (the optimal dendrimer/siRNA ratio) (**b**). The arrows point at instances of dendrimers as representative examples within the samples. A magnification of 100 000 × was used to analyze the complexes.

The morphological structure and size of the formed dendrimer/siRNA complex were explored using transmission electron microscopy (TEM) (Fig. 7). As a representative example, only the G3Si PEG6000 dendrimer was studied in detail. It was observed that the non-complexed dendrimer formed aggregates, while complexation with siRNA led to the dispersion of the dendrimer. Analysis of the transmission electron microscopy images revealed that the dendrimer G3Si PEG6000 had a spherical shape with a size of approximately 5.78 ± 1.19 nm.

**Figure 8:**
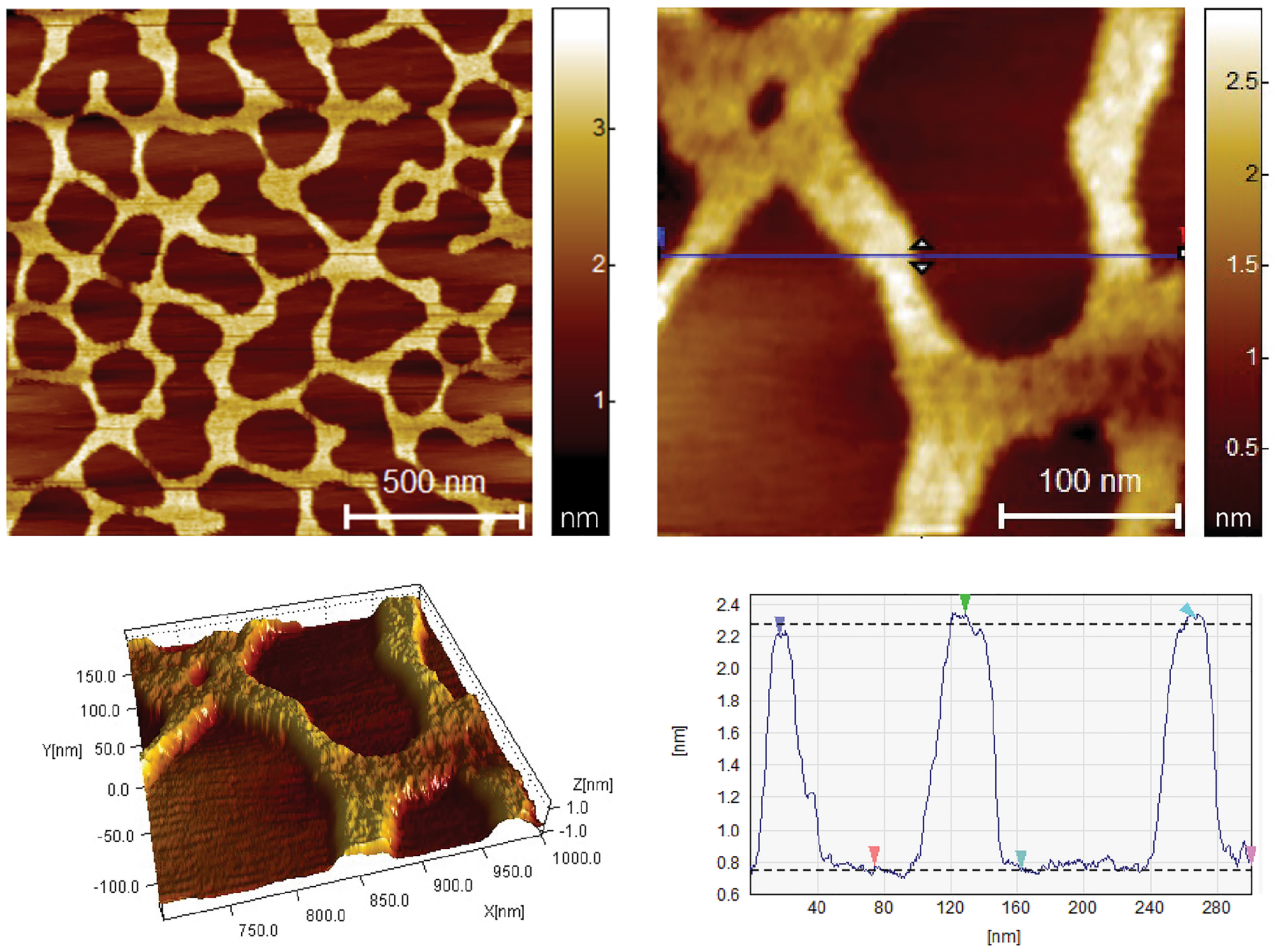
AFM images of siRNA and 3D visualization of its structure.

The morphological features and dimensional properties of the siRNA, the uncomplexed dendrimer and dendrimer/siRNA complex, were thoroughly examined using AFM. The AFM images of siRNA revealed distinctive arrangement patterns of siRNA molecules (Fig. 8). The structures exhibited a uniform height profile of approximately 0.77 nm, indicative of a well-organized arrangement consisting of 2 layers of siRNA molecules stacked on each other^25^. These measurements were reproducible across multiple analyses, affirming the reliability and consistency of the stacked siRNA conformation on the mica surface.

**Figure 9:**
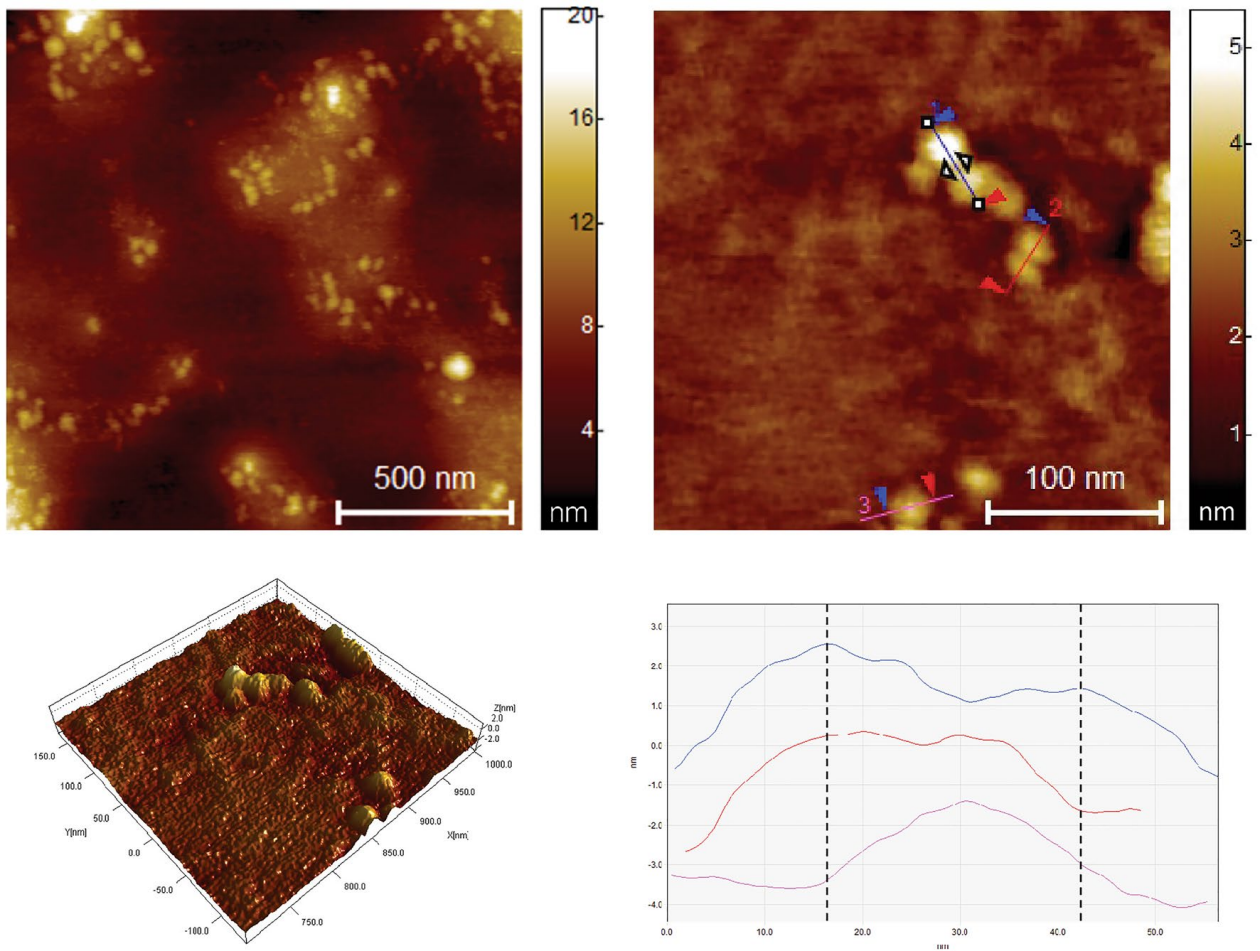
AFM images of G3Si PEG6000 dendrimer and 3D visualization of its structure.

The height images provided detailed information regarding the dendrimers' size distribution and spatial arrangement. The G3Si PEG6000 dendrimer exhibited an average diameter of approximately 22.5 nm. The size difference compared to the images of TEM is due to the interaction with the substrate, as well as flattening resulting from the in Tap- ping Mode (AFM) observation (Fig. 9).

**Figure 10:**
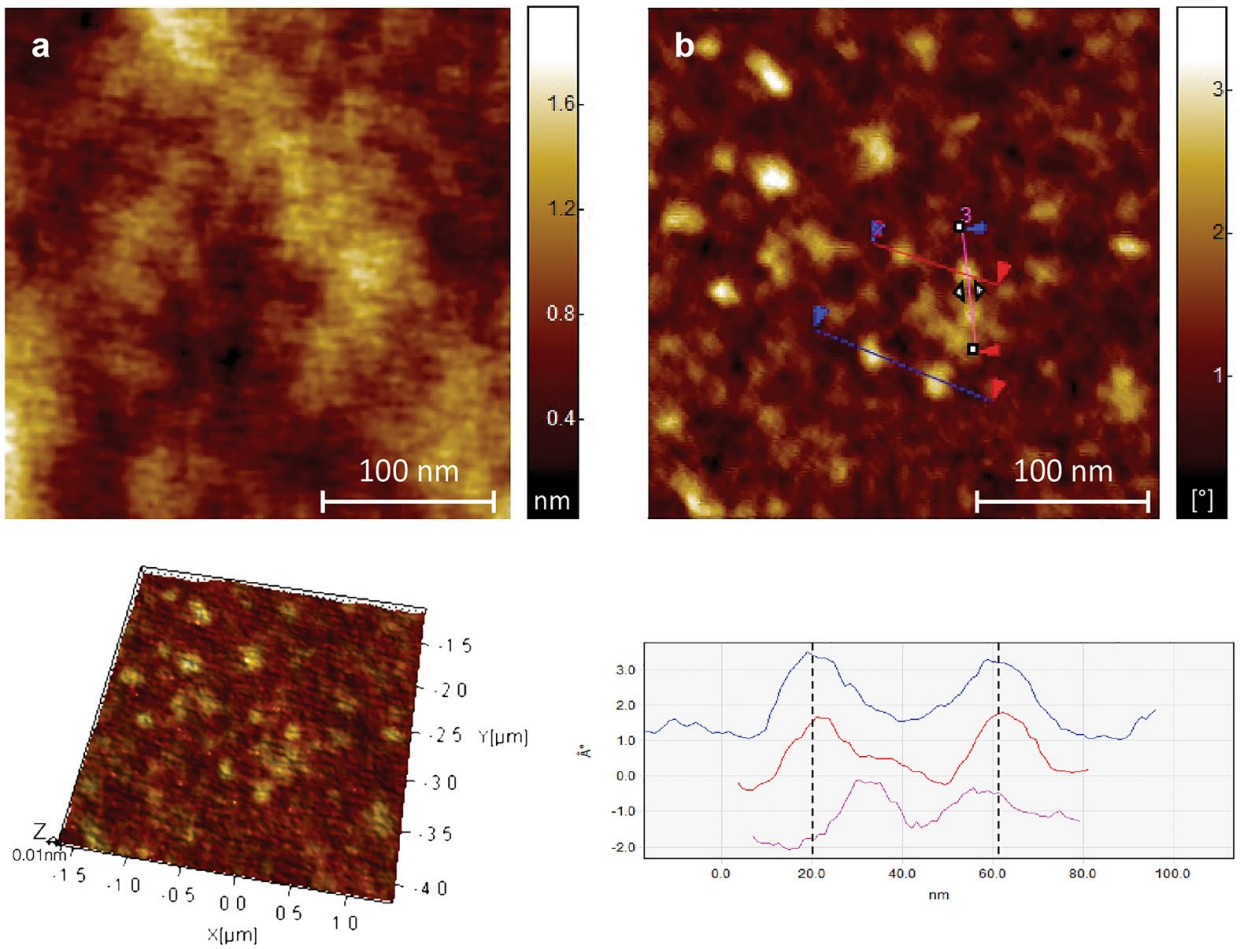
AFM height image (**a**) and phase image (**b**) of G3Si PEG6000/siRNA dendriplex in a molar ratio of 2.5:1 (the optimal dendrimer/siRNA ratio) and 3D visualization of its structure.

In order to assess the characteristics of the G3Si PEG6000/siRNA dendriplex, both height and phase images were acquired (Fig. 10). Our results demonstrated that the G3Si PEG6000/siRNA dendriplex exhibited an average diameter of approximately 14 nm.

### Fluorescence polarization

**Figure 11:**
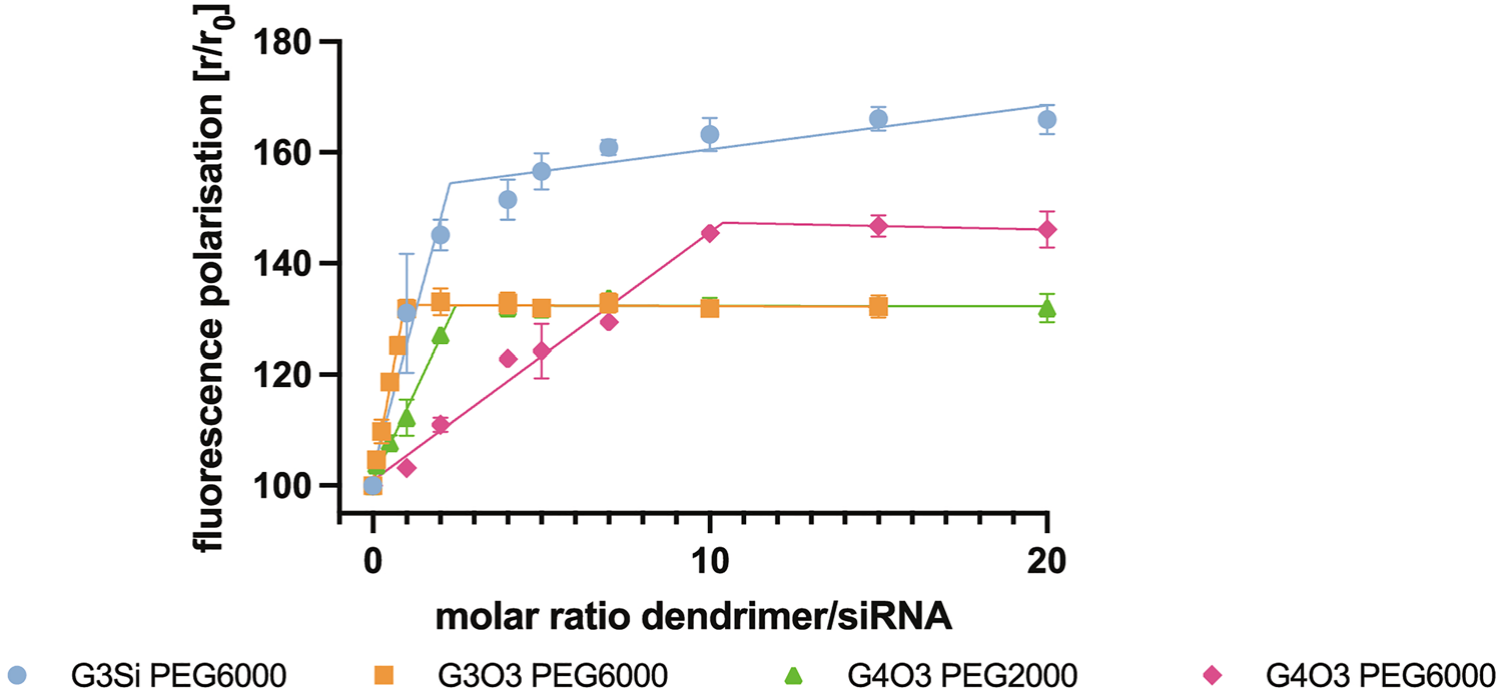
Changes in siRNA-FITC (1 μM) fluorescence polarization upon titration of the studied dendrimers. The excitation and emission wavelengths were 485 and 520 nm, accordingly. The results are shown for 3 repetitions as mean and standard deviation.

The fluorescence polarization of the evaluated dendrimers was analyzed and the results are presented in Fig. 11. The formation of dendrimer/siRNA complexes contributed to the reduction of the movement of siRNA molecules in suspension, which is observed as an increase in siRNA-FITC fluorescence polarization. During the addition of subsequent portions of tested dendrimers to siRNA-FITC solution, an increase in fluorescence polarization was observed until reaching the plateau phase which indicated the formation of the complexes.

### Time stability

**Figure 12:**
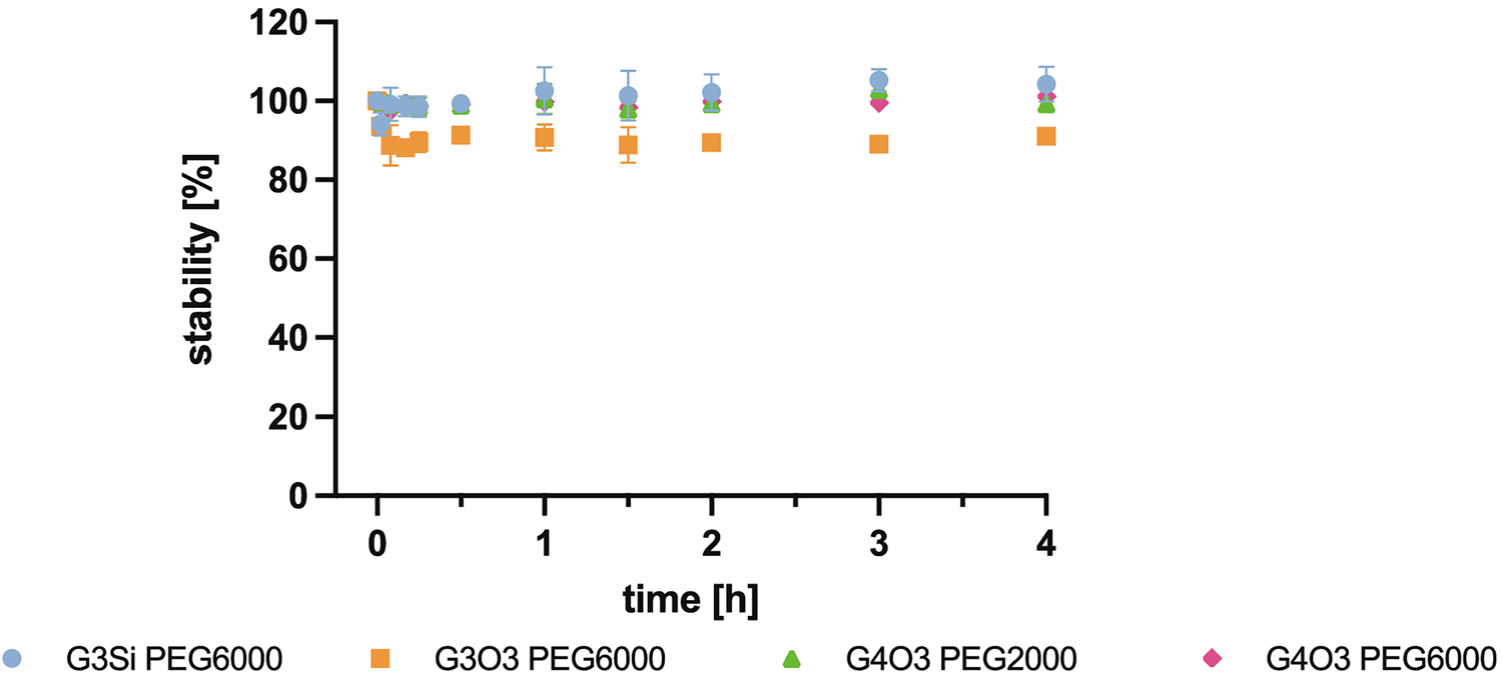
The time dependence of dendriplex fluorescence polarization, expressed as a percentage of control (siRNA-FITC without dendrimers). The results are shown for 3 repetitions as mean and standard deviation.

Measurement of the fluorescence polarization of the formed complexes dendrimer/siRNA-FITC at different times also allowed for the evaluation of their stability. The formed complexes (dendrimer/siRNA-FITC molar ratio calculated by fluorescence polarization measurement) were incubated and the fluorescence polarization was measured for 4 h. The obtained results (Fig. 12) showed that the fluorescence polarization of dendriplexes did not change significantly when increasing incubation time, proving that the formed complexes were stable over a 4 h period.

### Circular dichroism

**Figure 13:**
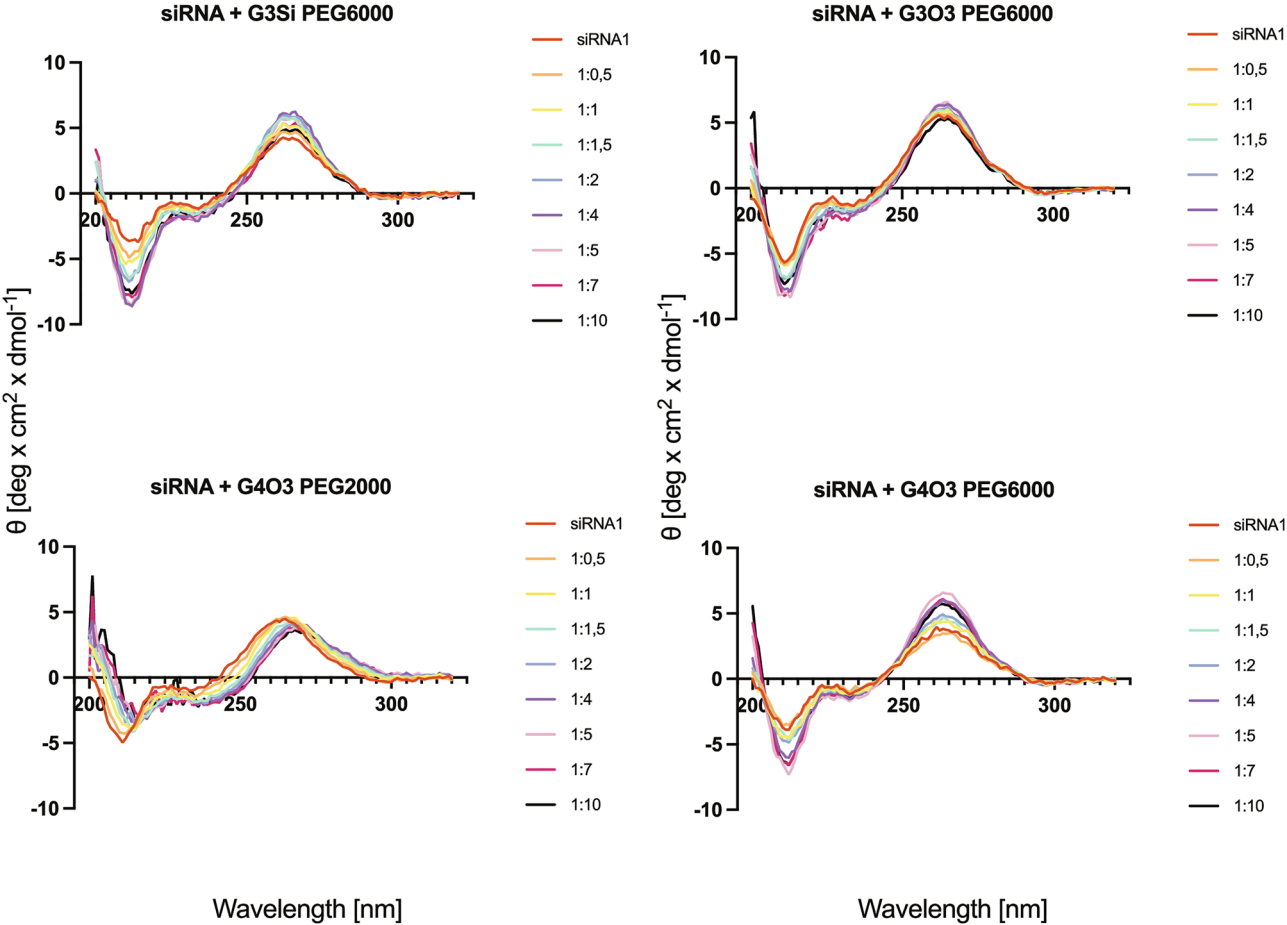
CD spectra for siRNA (C = 1 µM) in the presence of the studied dendrimers (C = 0.5–10 µM). The results are shown for 3 repetitions.

After normalization of ellipticity of the native siRNA spectra (the highest peak at 260 nm) at around 5 deg × cm^2^ × dmol^−1^, it was found that all tested dendrimers could bind to siRNA, changing the conformation (Fig. 13). The G3Si PEG6000, G3O3 PEG600 and G4O3 PEG6000 dendrimers caused a concentration-dependent increase in signal intensity for both the negative peak (205–215 nm) and the positive peak (260–270 nm) in the siRNA CD spectra. In contrast, increasing the concentration of G4O3 PEG2000 caused the flattening of both the negative and positive peaks. The spectral analysis revealed that the G4O3 PEG2000 induced a notable red-shift throughout the entire spectra.

**Figure 14:**
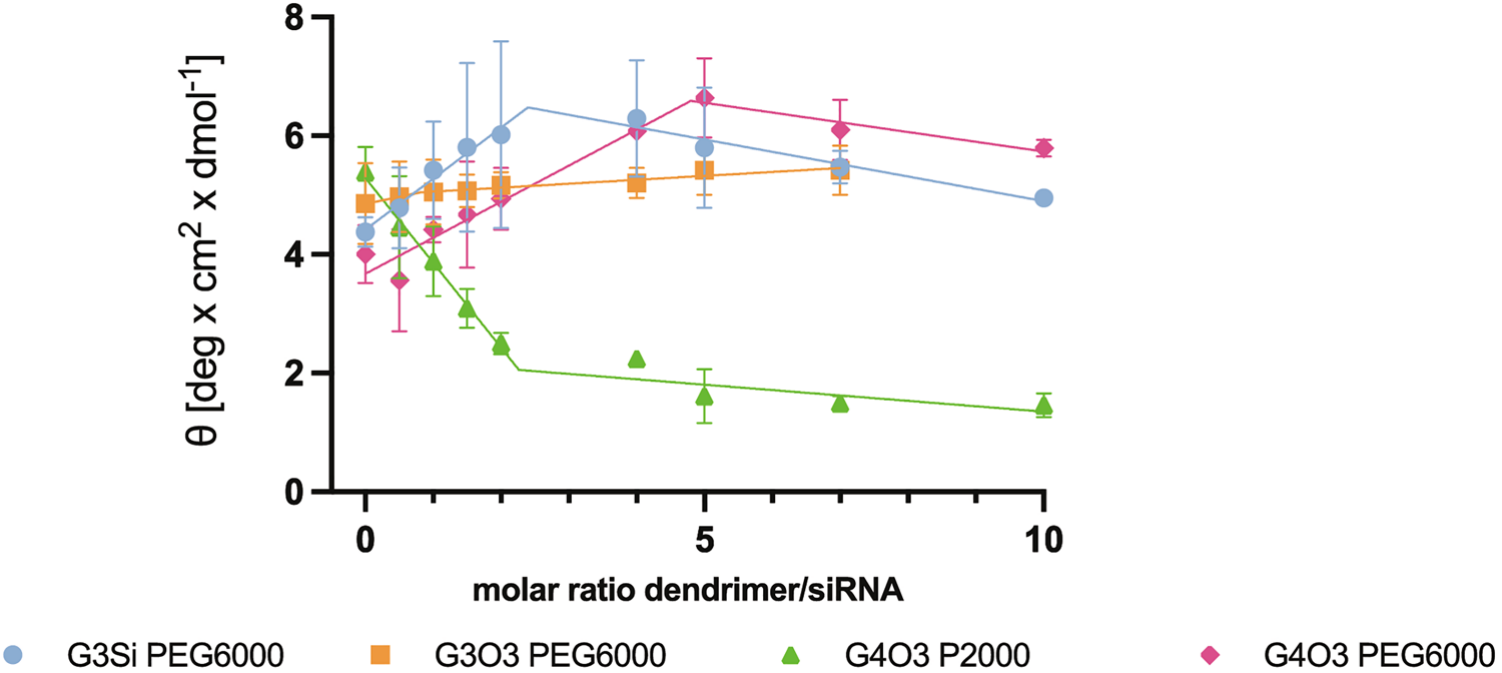
Changes in mean residue ellipticity of siRNA at λ equal to the dendrimers’ maximum peak (260–270 nm). The results are shown for 3 repetitions as mean and standard deviation.

The results indicate that the siRNA samples that were combined with the dendrimers G4O3 PEG6000 and G3Si PEG6000, respectively, exhibited the most substantial difference in ellipticity compared to other samples (Fig. 14).

### Gel electrophoresis

**Figure 15:**
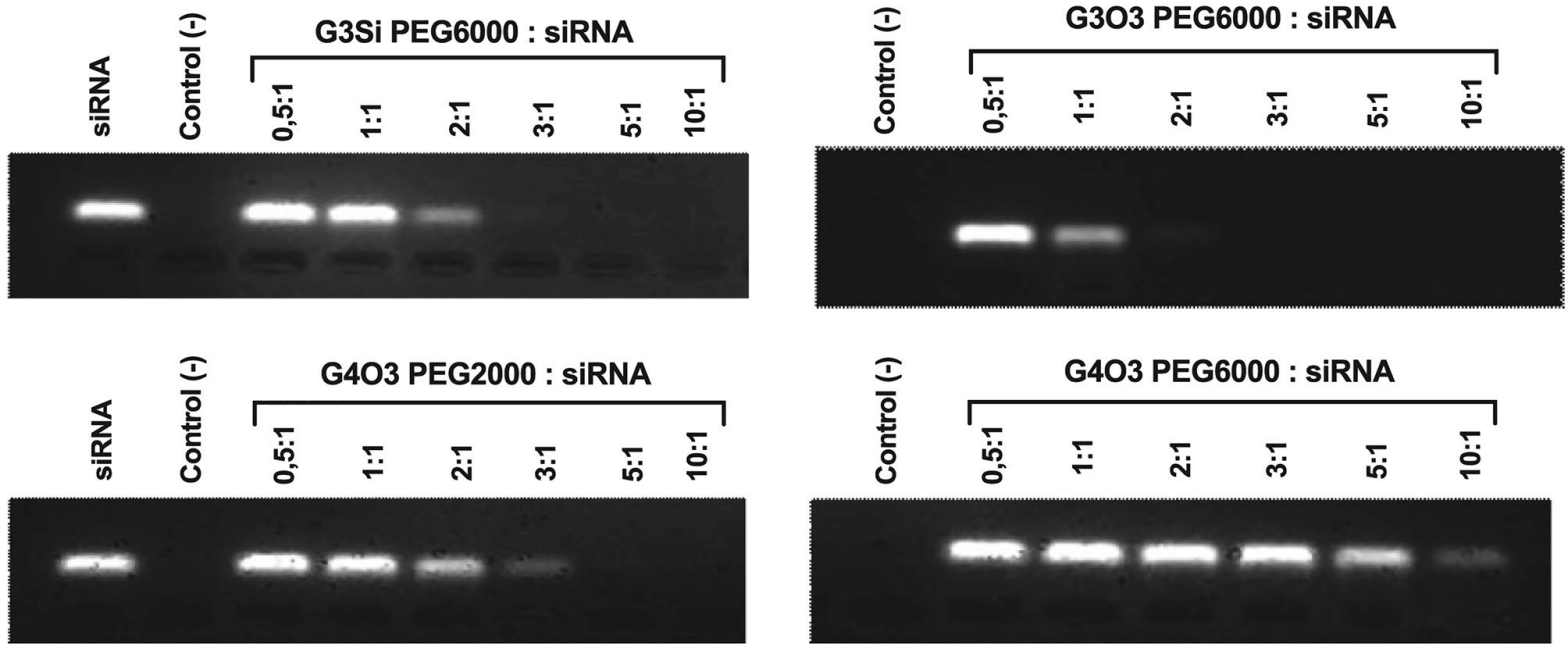
Analysis of dendrimer/siRNA complexes in different ratios in 3% agarose gel with GelRed. Complexes were prepared in 10 mmol/l PBS.

The visualization of siRNA electropherograms enabled the analysis of the dendrimer-siRNA complex formation. Gel electrophoresis results revealed that all dendrimers interacted with siRNA, leading to the quenching of the fluorescence signal. As the dendrimers formed complexes with siRNA, the amount of uncomplexed siRNA decreased until it was fully saturated and the fluorescence was completely extinguished. The data obtained suggested that out of the tested dendrimers, the G4O3 PEG6000 dendrimer formed complexes with siRNA that were optimally saturated in the highest molar ratio, while the G3O3 PEG6000 dendrimer exhibited the lowest molar ratio at which the complex with siRNA is optimally saturated (Fig. 15). The results obtained from all the biophysical assays describing the optimal saturation of the complexes are summarized in Table 3.

**Table 3:** The data collected from the conducted biophysical assays (excluding the gel electrophoresis) describing the optimal saturation of the complexes, were subjected to an analysis using segmented regression.

Dendrimer	Zeta potential	Fluorescence polarization	Circular dichroism	Gel electrophoresis*
G3Si PEG6000	2.52	2.29	2.40	Over 2
G3O3 PEG6000	4.93	0.94	0.82	Over 1
G4O3 PEG2000	2.67	2.46	2.27	Over 2
G4O3 PEG6000	9.03	10.39	4.80	Over 10

This analytical approach allowed for the identification of a change in slope, resulting in the identification of a sharp transition point. This rapid transition signified the ratio of the tested dendrimers to siRNA at which optimal saturation of the complex occurred. The table shows the molar ratio of the tested dendrimers to 1 mol of studied siRNA.

*Visual examination was used to evaluate the electropherograms obtained from the gel electrophoresis.

### Cytotoxicity

**Figure 16:**
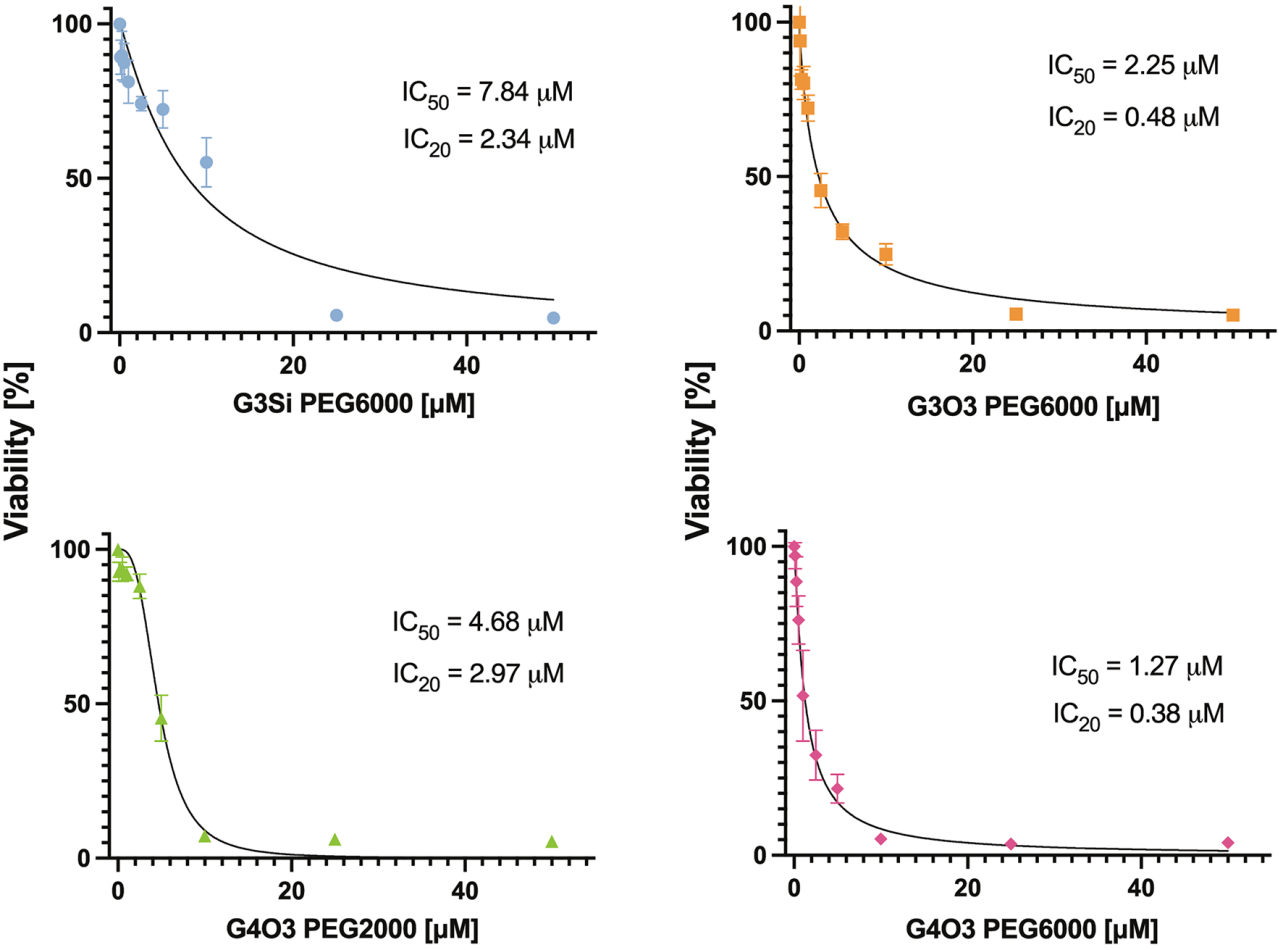
IC50 and IC20 values of HBEC-5i cells treated with dendrimers showed in MTT assay, after 24 h incubation. The results are shown for 6 repetitions as mean and standard deviation. The results are shown for 6 repetitions as mean and standard deviation.

An MTT assay was conducted to evaluate the cytotoxicity of studied dendrimers on cerebral microvascular endothelial cells (HBEC-5i). This assay measured mitochondrial oxidative activity of the HBEC-5i cells after incubation with dendrimers for 24 h. The findings of this study (Fig. 16) indicate that all dendrimers exhibited significant toxicity on HBEC-5i cell line at higher concentrations (> 25 μM). Among the tested dendrimers, G3Si PEG6000 exhibited the lowest IC50 (7.84 µM), with the dendrimers G4O3 PEG2000 and G3Si PEG6000 exhibiting the lowest IC20 (2.97 and 2.34 µM, respectively). G4O3 PEG6000 showed the highest toxicity at IC50 (1.27 µM) and IC20 (0.38 µM).

## Discussion

Since the discovery of the "cascade molecule" in 1978 and the first complete dendrimer family that was synthesized from generation 1 to 7 in 1985, nanoparticle development has led to the creation of various dendrimer structures with diverse applications in fields such as light harvesting and energy transfer, nanoscale catalysis, chemical sensors, unimolecular micelles, enzyme mimics, encapsulation of guest molecules, molecular recognition, diagnostic agents and gene and drug delivery^26–28^. Due to their unique structural features, dendrimers have received considerable attention for use as potential nucleic acid carriers^18,29–31^. Carbosilane dendrimers were found to be a stable and effective delivery system with high transfection efficiency by Weber et al.^28,32^ Advancing research in this area has the potential to overcome the current limitations and challenges of today’s therapy methods and unlock the full potential of dendrimers for the treatment of neurodegenerative disorders and other diseases. It is crucial to continue the development and characterization of these extraordinary tools for gene therapy applications.

The BBB is the most restrictive barrier within the body and prevents entry into the brain for most small molecules and nearly all macromolecular therapeutics developed for central nervous system (CNS) pathologies^33^. Highly astute scientific research must be pursued to achieve an applicable gene silencing directed against neurodegenerative disorders. The BBB presents obstacles to siRNA delivery to the CNS due to tight junctions preventing passive diffusion, low pinocytosis reducing active transport, and high metabolic activity leading to potential enzymatic degradation^34^. These features collectively impede the effective entry of therapeutic siRNA molecules into the brain. In addressing these challenges, our study delves into the intricate aspects of siRNA delivery. To address these challenges, we analyzed the biophysical characteristics of 4 dendrimers and their complexes with siRNA to identify a suitable candidate for a siRNA nanocarrier capable of transferring cargo safely across the BBB model. The findings of our study offer valuable insights into the process of complexation, stability in time, mechanisms of interaction, the influence of the tested dendrimers on the spatial structure of the oligonucleotide and the potential cytotoxic effects of the dendrimers on the human cerebral microvascular endothelium cells in the context of the challenges posed by crossing the BBB.

The size and surface charge of nano-delivery systems significantly impacts pharmacokinetics, absorption, tissue distribution and intracellular clearance. Therefore, careful consideration must be given to the selection of the appropriate size of carrier for the intended application. The BBB, which is characterized by tight intercellular junctions and zonula occludens between closely connected capillary endothelial cells, presents a formidable obstacle due to the non-fenestrated nature of brain capillaries^35,36^. Consequently, there is low probability of paracellular transport for nanoparticles compared to the transcytotic transport through cellular vesicles. However, it has been established that nanoparticles are able to exploit both the para-cellular and transcellular pathways^37^. Changes in the vasculature during neurodegenerative disorders, such as endothelial degeneration, can impact the normal diffusion of solutes across brain extracellular spaces, reducing the expression of tight and adherens junctions at the BBB, and ultimately affecting the delivery of therapeutic agents^38^. Although sole particle size cannot predict cellular absorption mechanisms, there is evidence that particles within certain size ranges utilize specific uptake pathways.

In our studies, dynamic light scattering (DLS), transmission electron microscopy (TEM) and atomic force microscopy (AFM) were used in the analysis of the size and shape of the dendrimers and dendriplexes. The particle size measurement obtained from DLS is referred to as a "hydrodynamic diameter", as it accounts for the core size of the nanoparticle and also for any polymers or molecules on the surface of the dendrimers. Consequently, DLS measurements exhibited larger mean diameters than those obtained by TEM and AFM due to factors such as particle aggregation, solvation shell, and PEG polymer^39^.

The AFM studies of the G3Si PEG6000 dendrimer showed a uniform particle size distribution. However, the size obtained from the AFM measurements differed from the results obtained from TEM. This difference may be attributed to the fact that the deposition of the dendrimer was performed on a strongly hydrophobic surface (mica). The dendrimer surface possesses highly charged ions exhibiting hydrophilic properties. On contact with hydrophobic air and solvent evaporation (water), significant deformation occurs, causing the dendrimer to assume a disk-like shape (Fig. 9). When we formed the G3Si PEG6000/siRNA dendriplex, the situation changed. Since the measurement is performed in a buffer system consisting of water and the siRNA dendrimer, we observed that after water evaporation the formed complex is likely located within the matrix, possibly the buffer and excess siRNA. The average size of the complex was approximately 14 nm. In this case, the complex size, more precisely its diameter, is determined by the attached siRNA. The reason for the complex being smaller than the non-complexed dendrimer can be attributed to the reduced impact of the environment (air) on the complex confined inside the matrix (Fig. 10).

TEM imaging of the non-complexed dendrimer showed that the G3Si PEG6000 dendrimer is approximately the same size and shape as haemoglobin, suggesting that its size is favourable for cellular uptake through the clathrin-independent endocytotic pathway. Particles in the submicron size range, such as the analyzed dendriplexes, have been found to use uptake pathways such as caveolae-mediated, clathrin-mediated endocytosis or macropinocytosis^17,36,40^. Multiple studies have demonstrated that the smaller nanocarriers exhibit a greater capacity to cross the BBB with an accelerated rate, and result in enhanced tissue penetration and deeper transport of their cargo^41,42^. As a consequence, small-sized dendrimers may have a greater ability to penetrate the BBB and achieve wider distribution throughout the tissue. The acquired data indicated that dendrimer G3Si PEG6000 possessed the smallest hydrodynamic diameter while also forming one of the smallest dendriplexes amongst those examined. It is also noteworthy that the smallest size of the G4O3 PEG6000 dendrimer compared to its less PEGylated counterpart (G4O3 PEG2000) may be attributed to greater hydrophobic interactions and increased polymer flexibility in highly PEGylated dendrimers. Additionally, high ionic strength modifies the observed zeta potential of the 2 dendrimers, as well as the PEG conformation and as result, affects complexation with nucleic acids^39,43–45^.

The positive surface charge of dendrimers and dendriplexes is a critical factor in determining their binding with other oppositely charged particles. It governs the stability of the complex, affects the interaction with the cell membrane and, ultimately, its transfection efficiency. However, a high positive surface charge may also result in membrane disruption via nano-hole formation, membrane thinning and erosion^46,47^. The charge of a nanoparticle's surface is expressed as the zeta potential, which is the electrostatic potential at the interface between the nanoparticles' diffuse layer of ions, called the shear plane, and the surrounding liquid. Among the tested dendrimers, the G3O3 PEG6000 and G4O3 PEG6000 exhibited the highest zeta potential. As anticipated, the dendrimers with the highest zeta potential exhibited the highest cytotoxicity. However, complexation with nucleic acid mitigates, to some extent, the cytotoxic effects of the dendrimer. Further studies are required to obtain a comprehensive picture of the cytotoxic effects of the studied carriers^47^. The positive surface charge of the dendrimers interacted with negatively charged phosphate molecules on the siRNA. As the electrostatic interactions are the predominant factor driving the complexation, the zeta potential enables the determination of the appropriate dendrimer concentration required for optimal complex saturation. The saturation point is achieved when the continuous addition of the dendrimer reaches a plateau indicating the optimal quantity of dendrimer molecules bound to the siRNA molecule. It was observed that dendrimer G4O3 PEG6000, despite having high zeta potential, creates saturated complexes with siRNA in relatively high concentrations. It is hypothesized that this may be due to high repulsion between the dendrimers^39^.

Various physicochemical methods were employed to investigate the efficacy of dendrimers in binding siRNAs and determining their optimal properties for forming a saturated complex. Among these methods was fluorescence polarization. Fluorescently labelled siRNA in solution at 20–37 °C has a flexible structure, but upon complexation, its rotation is hindered, fluorescence intensity lowered and polarization increased. The higher the polarization value, the stronger the siRNA is bound to a dendrimer^48^. Dendrimer G3Si PEG6000 reached the highest fluorescence polarization values. Thus, we conclude that the siRNA had the strongest affinity to this dendrimer. This was also confirmed by the fact that the dendrimer G3Si PEG6000 creates one of the smallest dendriplexes amongst all those tested. This may be due to the fact that the strong affinity of the siRNA to the dendrimer could result in tighter wrapping of the nucleic acid around the dendrimer molecules^17^. It is noteworthy that both G4O3 PEG6000 and G3Si PEG6000 dendrimers create dendriplexes of comparable size. Even though the dendriplex created with the G3Si PEG6000 dendrimer has a lower zeta potential than the dendriplex with G4O3 PEG6000 dendrimer, the former has higher fluorescence polarization. The G3Si PEG6000 dendrimer may induce a more significant effect on siRNA conformation while possessing a lower surface charge, most likely due to the geometry of peripheral cations and their availability for interaction with the siRNA. Fluorescence polarization was also employed to assess the time stability of the tested dendrimers. Notably, all the tested dendrimers displayed consistent fluorescence polarization values and no statistically significant changes were observed over the designated time period. This suggests that the dendrimers remained stable throughout the entire duration of the experiment. This consistent behaviour of the dendrimers over time further supports their potential.

Circular Dichroism (CD) was employed to investigate the impact of dendrimers on the 3-dimensional configuration of the siRNA. This technique is well-suited for studying nucleic acids due to the chiral nature resulting from the helical arrangement of nucleotides and the intrinsic asymmetry of nucleosides. Specifically, nucleic acids absorb left and right circularly polarized light unequally, leading to differential light absorption and conversion into elliptically polarized light. This conversion yields an ellipse with a characteristic angle, θ, whose tangent is equivalent to the ratio of the minor to major axes of the ellipse. The manifestation of ellipticity is commonly referred to as circular dichroism^49–51^. The absorption characteristic for nucleic acids is determined by a complex interplay of individual nucleotide absorption and their interactions. Therefore, the excitation coefficient of a nucleic acid at any given wavelength depends on its sequence and conformation^50^. The studied siRNA CD spectra exhibited a minimum near 210 nm, a maximum near 260 nm, and small negative CD values between 290 and 300 nm, indicating a right-handed A-form structure. However, the conformation of nucleic acids is dynamic and it changes upon exposure to dendrimers. The G3Si PEG6000, G3O3 PEG6000 and G4O3 PEG6000 dendrimers caused an increase in the ellipticity of the siRNA. Conversely, the G4O3 PEG2000 dendrimer decreased the ellipticity of the oligonucleotide. To investigate the interplay between dendrimers and nucleic acids further, it was necessary to consider the specific alterations occurring within the siRNA structure that influenced changes in spatial configuration and the resulting CD spectrum. Circular dichroism of nucleic acids is determined by the geometric relationship between coupled electronic transition dipoles on adjacent nucleotides and the distance between bases, with the stacking arrangement of the bases exerting a predominant influence on the overall CD signature. Based on these considerations, it can be inferred that the observed increase in siRNA ellipticity induced by the dendrimers was associated with hyperchromicity and manifested as an increase in absorption. Accordingly, the decrease of ellipticity caused by dendrimer G4O3 PEG2000 was related to hypochromicity and suggested the siRNA adopted a less compact arrangement with the dendrimer^49,50,52^. It is noteworthy that a red shift of the siRNA spectra was observed upon titration of dendrimer G4O2 PEG2000. This observation provided further evidence of conformational changes occurring in the oligonucleotide during complexation with the dendrimer^51,52^. The greatest difference in ellipticity was observed in the siRNA samples combined with dendrimer G4O3 PEG6000 and G3Si PEG6000, respectively. These findings suggested that these dendrimers exerted the most pronounced influence on the conformation of siRNA. The outcomes of this investigation aligned with those obtained via the fluorescence polarization assay. The analysis of CD spectra also exhibited a sufficient signal intensity, facilitating the identification of an optimal concentration of dendrimer required for complex saturation.

The formation of dendriplexes was investigated using a gel electrophoresis assay, resulting in the acquisition of presented electropherograms. As nucleic acid oligomers are polyanions at neutral pH, they migrated toward the positive electrode in the electrophoretic gels. Non-complexed siRNA traversing through the porous agarose gel stained with GelRed-stained created a fluorescent band. The formation of the studied dendrimer-siRNA complexes led to a reduction in fluorescence intensity, which we attributed to the hindrance of dye-siRNA interactions. As a result, the gradual decrease of band fluorescence was observed, with an increase of dendrimer concentration until a state of saturation was reached and led to complete fluorescence quenching. The results obtained from the gel electrophoresis and all other biophysical assays discussed are summarized in Table 2. The results are consistent with the results obtained by other methods.

To be utilized effectively, dendrimers must exhibit low toxicity and non-immunogenicity, as well as biocompatibility and biodegradability. These attributes are vital for ensuring safe and effective use in the development of various biomedical and pharmaceutical products. Cytotoxic properties of the tested dendrimers were assessed using a colourimetric assay measuring formazan, proportional to the number of viable cells with active mitochondrial dehydrogenases. Dendrimer G4O3 PEG6000 was found to have the highest cytotoxic properties among the dendrimers examined. G3Si PEG6000 and G4O3 PEG2000 dendrimers had the least cytotoxic effects on cells. Although G3Si PEG6000 and G4O3 PEG2000 exhibited comparable IC20 values, the cytotoxicity of G4O3 PEG2000 increased rapidly, resulting in a substantially reduced IC50 value. This finding raised concerns about the safety of G4O3 PEG2000 at higher concentrations or prolonged exposure. G3Si PEG6000 was a preferable option in terms of safety compared to G4O3 PEG2000, as it manifested a higher IC50 value and did not exhibit the rapid increase in cytotoxicity at higher concentrations. These results demonstrate the considerable variance in cytotoxicity among various dendrimers. The findings underscored the potential of G3Si PEG6000 as the superior nanocarrier for biomedical applications, both regarding safety while simultaneously revealing markedly elevated cytotoxic properties of the G4O3 PEG6000. It was found that dendrimers exhibiting the highest cytotoxicity also demonstrated the greatest zeta potential. This observation provided further evidence of the critical role played by the zeta potential in determining the biocompatibility and safety of dendrimers. It is essential to highlight that the amount of cationic quaternary ammonium groups (R-NMe_3_^+^) did not strictly correlate with zeta potential and cytotoxicity, as dendrimer G3Si PEG6000 did not have the least amount of cationic surface groups but exhibited the lowest zeta potential and cytotoxic properties. This confirms the significant impact of dendrimer composition and their structural characteristics.

The investigation conducted by Jiménez et al. used the 2G-NN16 carbosilane dendrimer for siRNA delivery to the brain, with similar cationic moieties as those presented in the current research. The findings of this study revealed that the tested dendrimers effectively transported siRNA to human normal astrocytes, resulting in gene silencing without inducing cytotoxicity at tested concentrations^53^. Dendrimers G3Si PEG600 and G4O3 PEG2000 did not possess cytotoxic properties, (viability lower than 80%), in concentrations of the 2G-NN16 dendrimer tested in the aforementioned study. It is important to highlight that the studies were performed on different cell lines. The same dendrimer, 2G-NN16, was utilized by Posadas et al. in a study demonstrating its transfection of siRNA into primary rat cortical neuronal cells. The study revealed that the 2G-NN16 dendrimer did not have significant cytotoxicity in all concentrations tested (up to 5 μM)^14^. The investigation by Serramía and colleagues, which employed carbosilane second-generation phloroglucinol-core dendrimers bearing R-NMe_3_^+^ moieties, also demonstrated the successful delivery of siRNA to normal human astrocytes. Notably, the study highlighted the transfection efficiency of these dendrimers in an in vivo mice model, thereby showing their potential for therapeutic interventions in various neurological disorders^54^. Another very interesting use of carbosilane dendrimers was performed by Białkowska et al. for the purpose of pro-apoptotic siRNA delivery to cancer cells. The cytotoxicity of 2 second generation dendrimers with tertiary or quaternary ammonium groups was tested with MCF-7 cells. Interestingly, the dendrimer with the tertiary ammonium groups appeared more cytotoxic. In their investigation, the team found that dendrimers created a saturated complex with siRNA at a dendrimer/siRNA ratio of 20:1. The resulting complexes exhibited higher levels of cytotoxicity compared to the uncomplexed dendrimers^55^. The studies described above emphasize the critical role of the dendrimer-to-siRNA ratio when developing dendrimer-based delivery systems for therapeutic use. These findings highlight the importance of carefully considering the optimal ratio of dendrimer to siRNA to achieve maximum efficacy while minimizing toxicity. Collectively, these studies demonstrate the vast potential of carbosilane dendrimers for versatile and efficient platforms for siRNA delivery and in other therapeutic applications. However, further research is necessary to optimize their design and ensure their safety and efficacy in clinical settings. These studies provide a strong foundation for future research in the field of dendrimer-based drug delivery and pave the way for the development of new and innovative therapeutic interventions.

## Conclusion

The present study involved the biophysical characterization of 4 carbosilane dendrimers and their dendriplexes with siRNA. Our findings provide insights into the complexation process, interaction mechanisms and influences of the dendrimers on the spatial structure of the oligonucleotide as well as the cytotoxic effect of the studied dendrimers on human brain endothelial cells. It was shown that the G4O3 PEG6000 dendrimer forms saturated complexes with siRNA at the least favourable dendrimer/siRNA ratio. It exhibited the highest cytotoxic activity among the tested compounds, rendering it unsuitable for further research. In contrast, the G3O3 PEG6000 dendrimer displayed a saturation point of the complex with the studied siRNA in a molar ratio of about 1/1, suggesting potential as an siRNA carrier. However, due to its high cytotoxicity and poor binding affinity with siRNA, it is not an ideal candidate despite the capacity to transport a greater amount of siRNA. The G3Si PEG6000 and G4O3 PEG2000 dendrimers exhibited optimal levels of dendriplex saturation in a similar molar ratio. The G4O3 PEG2000 dendrimer generates the largest dendriplexes of all the tested dendrimers. Moreover, this dendrimer displays a lower binding affinity towards siRNA and a higher cytotoxicity when compared to the G3Si PEG6000 dendrimer.

This research, therefore, suggests that the G3Si PEG6000 dendrimer is the optimal candidate for further study, as it exhibits the lowest cytotoxicity, the highest affinity for siRNA, exerts a pronounced influence on the conformation of siRNA, displays the smallest hydrodynamic diameter of a non-complexed dendrimer and has the smallest dendriplexes, all the while maintaining a good dendrimer concentration necessary for complex saturation.

While our research has provided valuable insights into the characteristics and potential of the G3Si PEG6000 dendrimers as a siRNA nanocarrier, further studies are crucial to fully understand its potential for biomedical application. Overall, the results of our study suggest a promising direction for further research, and we anticipate that continued efforts in this field will lead to significant advancements in siRNA delivery and disease treatment.

### Supplementary Information

Supplementary Information.

## Data Availability

The datasets generated during and/or analysed during the current study are available from the corresponding author on reasonable request. Correspondence and should be addressed to S.Z.

## References

[1] Griffith F (1928). The significance of pneumococcal types. J. Hyg..

[2] Friedmann T, Roblin R (1972). Gene therapy for human genetic disease?. Science.

[3] Ginn SL, Amaya AK, Alexander IE, Edelstein M, Abedi MR (2018). Gene therapy clinical trials worldwide to 2017: An update. J. Gene Med..

[4] Wirth T, Parker N, Ylä-Herttuala S (2013). History of gene therapy. Gene.

[5] Kim B, Park J, Sailor MJ (2019). Rekindling RNAi therapy: Materials design requirements for in vivo siRNA delivery. Adv. Mater..

[6] Xu H, Li Z, Si J (2014). Nanocarriers in gene therapy: A review. J. Biomed. Nanotechnol..

[7] Hudry E, Vandenberghe LH (2019). Therapeutic AAV gene transfer to the nervous system: A clinical reality. Neuron.

[8] Ramamoorth M (2015). Non viral vectors in gene therapy—An overview. JCDR.

[9] Hollon T (2000). Researchers and regulators reflect on first gene therapy death. Nat. Med..

[10] Waehler R, Russell SJ, Curiel DT (2007). Engineering targeted viral vectors for gene therapy. Nat. Rev. Genet..

[11] Woods N-B (2003). Lentiviral vector transduction of NOD/SCID repopulating cells results in multiple vector integrations per transduced cell: Risk of insertional mutagenesis. Blood.

[12] Nayerossadat N, Ali P, Maedeh T (2012). Viral and nonviral delivery systems for gene delivery. Adv. Biomed. Res..

[13] Haensler J, Szoka FC (1993). Polyamidoamine cascade polymers mediate efficient transfection of cells in culture. Bioconjugate Chem..

[14] Posadas I (2009). Highly efficient transfection of rat cortical neurons using carbosilane dendrimers unveils a neuroprotective role for HIF-1α in early chemical hypoxia-mediated neurotoxicity. Pharm. Res..

[15] Abbasi E (2014). Dendrimers: Synthesis, applications, and properties. Nanoscale Res. Lett..

[16] Svenson S, Tomalia DA (2005). Dendrimers in biomedical applications—Reflections on the field. Adv. Drug Deliv. Rev..

[17] Peng S-F (2010). Effects of the nanostructure of dendrimer/DNA complexes on their endocytosis and gene expression. Biomaterials.

[18] Santander-Ortega MJ, Lozano MV, Uchegbu IF, Schätzlein AG, Narain R (2016). 6—Dendrimers for gene therapy. Polymers and Nanomaterials for Gene Therapy.

[19] Hong S (2004). Interaction of poly(amidoamine) dendrimers with supported lipid bilayers and cells: Hole formation and the relation to transport. Bioconjugate Chem..

[20] Leroueil PR (2008). Wide varieties of cationic nanoparticles induce defects in supported lipid bilayers. Nano Lett..

[21] Brandon JA, Farmer BC, Williams HC, Johnson LA (2018). APOE and Alzheimer’s disease: Neuroimaging of metabolic and cerebrovascular dysfunction. Front. Aging Neurosci..

[22] Strašák T (2012). Carbosilane metallodendrimers with titanocene dichloride end groups. Organometallics.

[23] Sánchez-Nieves J, Ortega P, Muñoz-Fernández MA, Gomez R, Mata F (2010). Synthesis of carbosilane dendrons and dendrimers derived from 1,3,5-trihydroxybenzene. Tetrahedron.

[24] Mosmann T (1983). Rapid colorimetric assay for cellular growth and survival: Application to proliferation and cytotoxicity assays. J. Immunol. Methods.

[25] Zhang C (2006). siRNA-containing liposomes modified with polyarginine effectively silence the targeted gene. J. Control. Release.

[26] Buhleier E, Wehner W, Vögtle F (2002). Cascade’- and ‘nonskid-chain-like’ syntheses of molecular cavity topologies. Synthesis.

[27] Tomalia DA (1985). A new class of polymers: starburst-dendritic macromolecules. Polym. J..

[28] Bermejo JF (2007). Water-Soluble Carbosilane Dendrimers: Synthesis Biocompatibility and Complexation with Oligonucleotides; Evaluation for Medical Applications. Chem. Eur. J..

[29] Dufès C, Uchegbu IF, Schätzlein AG (2005). Dendrimers in gene delivery. Adv. Drug Deliv. Rev..

[30] Boas U, Heegaard PMH (2004). Dendrimers in drug research. Chem. Soc. Rev..

[31] Biswas S, Torchilin V (2013). Dendrimers for siRNA Delivery. Pharmaceuticals.

[32] Weber N (2008). Characterization of carbosilane dendrimers as effective carriers of siRNA to HIV-infected lymphocytes. J. Control Release.

[33] Pardridge WM (2005). The blood-brain barrier: Bottleneck in brain drug development. NeuroRx.

[34] Kimura S, Harashima H (2020). Current status and challenges associated with CNS-targeted gene delivery across the BBB. Pharmaceutics.

[35] Shilo M (2015). The effect of nanoparticle size on the probability to cross the blood-brain barrier: An in-vitro endothelial cell model. J Nanobiotechnology.

[36] Danaei M (2018). Impact of particle size and polydispersity index on the clinical applications of lipidic nanocarrier systems. Pharmaceutics.

[37] Hersh DS (2016). Evolving Drug delivery strategies to overcome the blood brain barrier. Curr. Pharm. Des..

[38] Sweeney MD, Sagare AP, Zlokovic BV (2018). Blood–brain barrier breakdown in Alzheimer disease and other neurodegenerative disorders. Nat. Rev. Neurol..

[39] Rabanel J-M, Hildgen P, Banquy X (2014). Assessment of PEG on polymeric particles surface, a key step in drug carrier translation. J. Control. Release.

[40] Conner SD, Schmid SL (2003). Regulated portals of entry into the cell. Nature.

[41] Hanada S (2014). Cell-based in vitro blood-brain barrier model can rapidly evaluate nanoparticles’ brain permeability in association with particle size and surface modification. Int. J. Mol. Sci..

[42] Gao K, Jiang X (2006). Influence of particle size on transport of methotrexate across blood brain barrier by polysorbate 80-coated polybutylcyanoacrylate nanoparticles. Int. J. Pharm..

[43] Hashemi M (2015). PEGylation of polypropylenimine dendrimer with alkylcarboxylate chain linkage to improve DNA delivery and cytotoxicity. Appl. Biochem. Biotechnol..

[44] Martinho N (2014). Molecular modeling to study dendrimers for biomedical applications. Molecules.

[45] Andrian T, Pujals S, Albertazzi L (2021). Quantifying the effect of PEG architecture on nanoparticle ligand availability using DNA-PAINT. Nanoscale Adv..

[46] Yang J, Zhang Q, Chang H, Cheng Y (2015). Surface-engineered dendrimers in gene delivery. Chem. Rev..

[47] Kesharwani P (2015). PAMAM dendrimers as promising nanocarriers for RNAi therapeutics. Mater Today.

[48] Shcharbin D, Pedziwiatr E, Bryszewska M (2009). How to study dendriplexes I: Characterization. J. Control. Release.

[49] Ranjbar B, Gill P (2009). Circular dichroism techniques: Biomolecular and nanostructural analyses—A review. Chem. Biol. Drug Des..

[50] Bloomfield V, Crothers D, Tinoco I (2000). Nucleic Acids: Structures, Properties, and Function.

[51] Fasman GD (1996). Circular Dichroism and the Conformational Analysis of Biomolecules.

[52] Chang PKC, Prestidge CA, Bremmell KE (2017). Interfacial analysis of siRNA complexes with poly-ethylenimine (PEI) or PAMAM dendrimers in gene delivery. Colloids Surf. B Biointerfaces.

[53] Jiménez JL (2010). Carbosilane dendrimers to transfect human astrocytes with small interfering RNA targeting human immunodeficiency virus. BioDrugs.

[54] Serramía MJ (2015). In vivo delivery of siRNA to the brain by carbosilane dendrimer. J. Control. Release.

[55] Białkowska K (2021). Interaction of cationic carbosilane dendrimers and their siRNA complexes with MCF-7 cells. Int. J. Mol. Sci..

