# Supplementary material for: Synthesis and biophysical evaluation of carbosilane dendrimers as therapeutic siRNA carriers
Source: Sci Rep. 2024 Jan 18;14:1615. doi: 10.1038/s41598-024-51238-w (PMC10796380; doi:10.1038/s41598-024-51238-w)
Supplement: Supplementary file 1 — Supplementary Information. [file 41598_2024_51238_MOESM1_ESM.docx]

# Supplementary information for:

# Synthesis and Biophysical Evaluation of Carbosilane Dendrimers as Therapeutic siRNA Carriers

Serafin Zawadzki ^1,2^, Ángela Martín-Serrano ^3^, Elżbieta Okła ^1^, Marta Kędzierska ^1^, Sandra Garcia-Gallego ^3,4,5^, Paula O. López ^3,4,5^, Francisco J. de la Mata ^3,4,5^, Sylwia Michlewska ^6^, Tomasz Makowski ^7^, Maksim Ionov ^1,8^, Elżbieta Pędziwiatr-Werbicka ^1^, Maria Bryszewska ^1^ and Katarzyna Miłowska ^1^

1. Department of General Biophysics, Faculty of Biology and Environmental Protection, University of Lodz, 141/143 Pomorska St., 90-236 Lodz, Poland; 2. BioMedChem Doctoral School of the University of Lodz and Lodz Institutes of the Polish Academy of Sciences, 21/23 Matejki St., 90-237 Lodz, Poland; 3. University of Alcalá, Department of Organic and Inorganic Chemistry, IQAR, 28805 Madrid, Spain; 4. Networking Research Center on Bioengineering, Biomaterials and Nanomedicine (CIBER-BBN), 28029 Madrid, Spain; 5. Ramón y Cajal Health Research Institute (IRYCIS), 28034 Madrid, Spain; 6. Laboratory of Microscopic Imaging and Specialized Biological Techniques, Faculty of Biology and Environmental Protection, University of Lodz, Banacha 12/16, 90-237 Lodz, Poland; 7. Centre of Molecular and Macromolecular Studies, Polish Academy of Sciences, Sienkiewicza 112, 90-363 Lodz, Poland; 8. Mazovian Academy in Plock, Collegium Medicum, Faculty of Medicine, 2 Dabrowskiego Sq, 09-402 Plock, Poland

**Figure S1. a)** ^1^H-NMR (CDCl_3_) of intermediate compound G_3_Si(SPEG6000)(SNMe_2_HCl)_31_. The signal at 3,60 ppm corresponds to PEG and NMe_2·_HCl, which are overlapped. **b)** ^1^H-NMR (CDCl_3_) of intermediate compound G_3_Si(SPEG6000)(SNMe_2_)_31_. The signal at 3,60 ppm corresponds to PEG and the signal at 2,25 ppm corresponds to NMe_2_

**Figure S2.** ^1^H-NMR (DMSO) of the final compounds. The signal at 3.40 ppm corresponds to PEG and NMe_3_, which are overlapped.


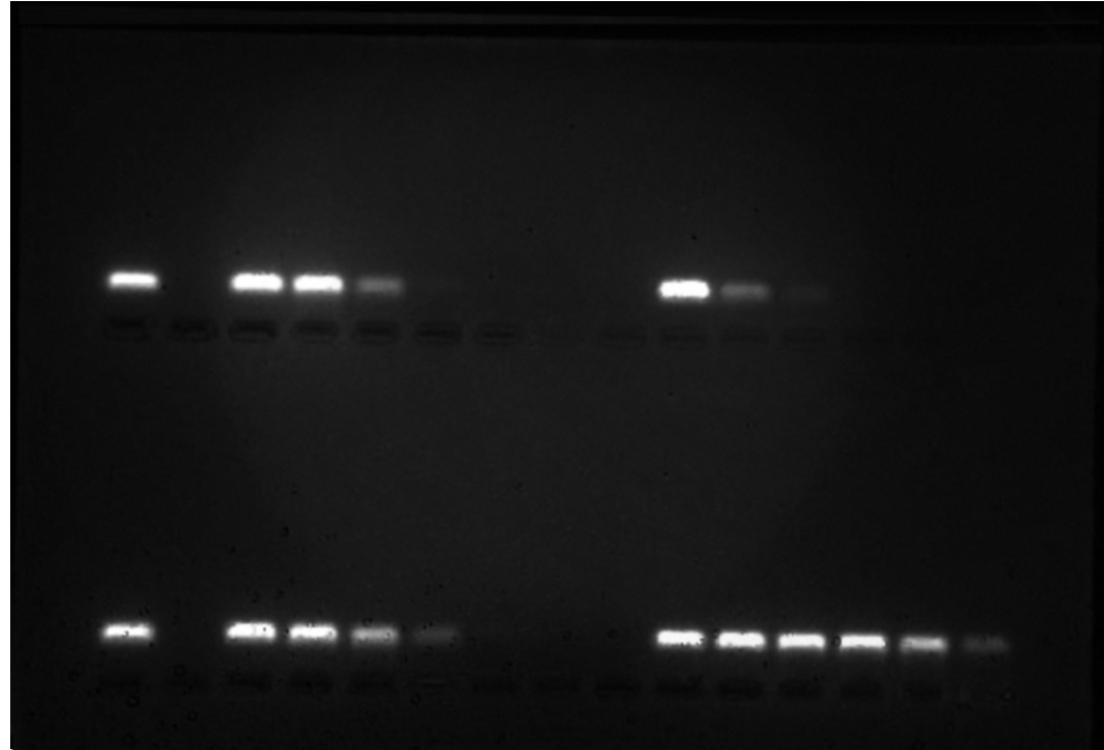


**Figure S3.** Original image corresponding to the Figure 15 in the main manuscript. Analysis of dendrimer/siRNA complexes in different ratios in 3% agarose gel with GelRed. Complexes were prepared in 10 mmol/l PBS.
